# Optical Sensor-Based Approaches in Obesity Detection: A Literature Review of Gait Analysis, Pose Estimation, and Human Voxel Modeling

**DOI:** 10.3390/s25154612

**Published:** 2025-07-25

**Authors:** Sabrine Dhaouadi, Mohamed Moncef Ben Khelifa, Ala Balti, Pascale Duché

**Affiliations:** J-AP2S “Laboratoire Jeunesse-Activité Physique et Sportive-Santé”, Université de Toulon, Campus La Garde, 83062 Toulon, France; sabrine-dhaouadi@etud.univ-tln.fr (S.D.); ala.balti.esstt@gmail.com (A.B.); pascale.duche@univ-tln.fr (P.D.)

**Keywords:** optical sensors, obesity detection, gait analysis, pose estimation, human voxel modeling

## Abstract

**Highlights:**

**What are the main findings?**

- This review examines optical and vision-based sensors including pose estimation (OpenPose, MediaPipe), infrared depth sensing, and 3D body modelling for non-contact obesity detection through gait and posture analysis.
- It highlights AI-driven, real-time capabilities and addresses challenges such as measurement accuracy, environmental factors, scalability, and ethical concerns (privacy, consent, algorithmic bias). Hybrid sensor approaches are proposed to improve robustness.

**What is the implication of the main finding?**

- The findings show strong potential of AI-driven, contactless sensors to improve obesity screening and personalized monitoring beyond traditional static methods.
- Successful translation into practice requires addressing technical and ethical issues to ensure equitable, reliable, and scalable adoption in healthcare and public health.

**Abstract:**

Optical sensor technologies are reshaping obesity detection by enabling non-invasive, dynamic analysis of biomechanical and morphological biomarkers. This review synthesizes recent advances in three key areas: optical gait analysis, vision-based pose estimation, and depth-sensing voxel modeling. Gait analysis leverages optical sensor arrays and video systems to identify obesity-specific deviations, such as reduced stride length and asymmetric movement patterns. Pose estimation algorithms—including markerless frameworks like OpenPose and MediaPipe—track kinematic patterns indicative of postural imbalance and altered locomotor control. Human voxel modeling reconstructs 3D body composition metrics, such as waist–hip ratio, through infrared-depth sensing, offering precise, contactless anthropometry. Despite their potential, challenges persist in sensor robustness under uncontrolled environments, algorithmic biases in diverse populations, and scalability for widespread deployment in existing health workflows. Emerging solutions such as federated learning and edge computing aim to address these limitations by enabling multimodal data harmonization and portable, real-time analytics. Future priorities involve standardizing validation protocols to ensure reproducibility, optimizing cost-efficacy for scalable deployment, and integrating optical systems with wearable technologies for holistic health monitoring. By shifting obesity diagnostics from static metrics to dynamic, multidimensional profiling, optical sensing paves the way for scalable public health interventions and personalized care strategies.

## 1. Introduction

### 1.1. Background and Motivation

Obesity has emerged as a critical global health concern, affecting individuals across all age groups and geographic regions. In 2022, more than 1 billion people worldwide—equivalent to one in eight—were living with obesity, with adult rates more than doubling and childhood and adolescent rates quadrupling since 1990 [1,2]. If current trends continue, projections using 2023 as the reference year indicate that over half of the global population will be overweight or obese by 2035, with obesity alone expected to rise from 14% to 24%, affecting nearly 2 billion people. Regionally, adult obesity is projected to reach 47–49% in the Americas, exceed 40% among women in the Eastern Mediterranean, reach 35% of women and 39% of men in Europe, and double in South East Asia—from 4% to 10% in men and from 8% to 16% in women [2,3]. Notably, the increase in obesity is steepest among children and adolescents: the percentage of boys affected is projected to double from 10% to 20% and girls from 8% to 18% between 2020 and 2035 [3]. The burden is not limited to high-income countries; rapid increases are also observed in low- and middle-income nations, particularly in Asia and Africa, where childhood overweight rates have surged by nearly 24% since 2000 [4].

Obesity is a multifactorial chronic disease with complex physiological, psychological, and socioeconomic implications. It is a major risk factor for non-communicable diseases, including cardiovascular disease, type 2 diabetes, osteoarthritis, and several cancers [1,2]. The economic impact is substantial, with annual global healthcare costs attributable to obesity exceeding USD 2 trillion [3]. Despite a strong evidence base for effective interventions, implementation remains patchy, and the epidemic continues to escalate [1].

Early detection and intervention are paramount, as obesity often leads to progressive impairment in physical function, quality of life, and long-term health outcomes [1,2]. Traditional screening methods, such as body mass index (BMI), provide only a static snapshot and may not capture the early biomechanical changes linked to excess weight. With time passed without early detection, this can cause musculoskeletal problems such as genu valgum or genu varum. Increasing evidence highlights the importance of functional markers especially those related to movement and gait as early indicators of obesity-related health risks [5,6].

### 1.2. Gait as a Diagnostic Tool

Human gait is a complex, dynamic process that reflects the integration of neuromuscular, skeletal, and metabolic systems. In the context of obesity, gait analysis has emerged as a valuable tool for identifying early biomechanical alterations that precede clinical symptoms [5]. Obese individuals—both adults and children—consistently demonstrate distinct gait characteristics: reduced stride length, slower walking speed, increased double support time, and greater asymmetry in joint loading, particularly at the hip, knee, and ankle [5]. These changes are not merely compensatory responses to increased body mass; they are also predictive of future musculoskeletal complications, reduced mobility, and diminished quality of life.

Recent studies using inertial measurement units (IMUs) and deep learning models have shown that gait patterns can accurately differentiate between normal-weight and obese adolescents, achieving classification accuracies as high as 97% [6]. Obese individuals exhibit shorter step lengths, slower speeds, and greater variability in gait, supporting the use of gait metrics as sensitive markers for early detection and monitoring of obesity-related functional decline [5,6]. Unlike static measures such as BMI or waist circumference, gait analysis provides a dynamic assessment of how excess weight affects daily movement and joint stress.

However, traditional gait analysis methods—such as marker-based motion capture systems and force plates—are often expensive, time-consuming, and limited to specialized laboratories. These constraints have historically restricted the use of gait analysis in routine clinical or community-based screening.

### 1.3. Shift in Technology: Toward Optical and Computational Sensing

Technological advances over the past decade have transformed the landscape of biomechanical assessment. Optical sensor systems, including RGB-D cameras (e.g., Microsoft Kinect, Intel RealSense), stereo vision, and monocular camera setups, now enable robust, markerless motion capture in real-world environments. These systems, when integrated with artificial intelligence (AI) and machine learning algorithms, allow for the extraction of detailed gait and posture metrics from simple video or depth data, making large-scale, non-invasive health screening feasible and cost-effective.

Markerless pose estimation frameworks—such as OpenPose, MediaPipe, and HRNet—can extract 2D or 3D skeletal keypoints from video input in real time, enabling efficient analysis of joint trajectories, angles, and coordination. These tools have been successfully applied to detect gait abnormalities in a range of clinical populations, including those with neurological and metabolic disorders, and are now being adapted for obesity screening. Additionally, 3D voxel modeling techniques derived from multi-view images or depth data provide volumetric insights into body composition, posture, and load distribution factors, highly relevant to obesity diagnosis and monitoring.

The integration of AI-powered analysis with optical sensing offers several advantages:

- Non-invasiveness: No physical contact or markers required, increasing user comfort and compliance.
- Scalability: Portable and low-cost systems enable deployment in diverse settings, from clinics to homes and schools.
- Automation: AI-driven pipelines facilitate rapid, objective assessment, reducing operator dependency and human error.
- Personalization: Continuous monitoring allows for individualized feedback and early intervention.

Despite these advances, challenges remain. There are ongoing debates regarding the reliability and validity of markerless optical systems compared to gold-standard laboratory instrumentation. Most algorithms are trained on normative datasets with limited representation of obese or morphologically diverse individuals, raising concerns about generalizability and algorithmic bias. Technical issues such as occlusion, clothing variability, and limited ground-truth data further complicate validation and deployment.

### 1.4. Scope and Objectives of the Review

Given these developments, the present review aims to consolidate and critically evaluate current optical sensor-based approaches for obesity detection, with a focus on three key domains:

- Optical gait analysis systems that derive spatiotemporal and kinematic metrics from video or depth data.
- Vision-based pose estimation frameworks that infer body mechanics from 2D/3D skeletal reconstructions.
- Three-dimensional voxel modeling techniques that provide volumetric insights into posture and body shape relevant to obesity diagnosis.

This review is intended for a multidisciplinary audience, including researchers and developers in biomedical sensing, artificial intelligence, health technology, biomechanics, and clinical diagnostics. By synthesizing findings from recent literature, we have the following aims:

- Provide an accessible overview of state-of-the-art methodologies and comparative system performance.
- Discuss validation, accessibility, and ethical considerations in deploying these technologies.
- Highlight both the current potential and limitations of optical sensor-based systems.
- Identify opportunities for future research and clinical translation.

In conclusion, the integration of gait analysis, pose estimation, and voxel modeling through optical sensing technologies holds transformative promise for early, individualized, and scalable obesity diagnostics. This is particularly significant for children and adolescents, where early detection and intervention can have lifelong health benefits. By bridging advances in sensing technology and AI with clinical needs, the field is poised to make a substantial impact on global efforts to curb the obesity epidemic.

## 2. Review Methodology

This literature review was conducted in accordance with the PRISMA (Preferred Reporting Items for Systematic Reviews and Meta-Analyses) 2020 guidelines. While this article is not a formal systematic review, we adhered to PRISMA principles to ensure methodological rigor and reproducibility. The primary research question guiding this review was expanded to encompass multiple dimensions:

*“What are the current optical sensor technologies and methodological approaches used for detecting and analyzing obesity through gait analysis, pose estimation, and human voxel modeling?”*

Secondary questions include the following:

1. *How has the landscape of optical sensor technology for obesity detection evolved since 2000?*
2. *What are the comparative advantages of different optical sensing modalities for obesity assessment?*
3. *What methodological challenges exist in validating these technologies across diverse populations?*
4. *How do optical sensor approaches compare with traditional obesity assessment methods?*

We included studies published in English prioritizing those with significant academic influence (e.g., citation frequency, high-impact venues) to ensure methodological robustness and relevance.

### 2.1. Search Strategy and Information Sources

As shown in Table 1, a comprehensive search was conducted using seven primary electronic databases to ensure wide coverage across medical, engineering, and computer science domains:

The search period covered January 2000 through April 2025, capturing both foundational works and recent technological advances. Additionally, we employed citation tracking (both forward and backward) to identify seminal papers that may have been missing in the database searches.

The search strategy employed controlled vocabulary (MeSH terms where appropriate) combined with free-text keywords using Boolean operators, field limiters, and truncation. Search strings were developed using the PICOS framework (Population, Intervention, Comparison, Outcomes, Study design) and refined through iterative testing. Table 2 presents the detailed search strategy developed for PubMed, which was subsequently adapted for other databases.

The final search combined these concepts using appropriate Boolean operators (AND, OR) and was adapted to each database’s specific syntax requirements.

The selection process consisted of three distinct phases:

✓ Initial Screening: Two independent reviewers screened titles and abstracts against the inclusion/exclusion criteria using Rayyan software to manage the screening process. Disagreements were resolved through discussion or by consulting a third reviewer when necessary.
✓ Full-Text Assessment: Full texts of potentially eligible studies were retrieved and independently assessed by two reviewers. A standardized form was used to document reasons for exclusion.
✓ Final Selection: The final set of included studies was determined after resolving all disagreements through consensus meetings.

Inter-rater reliability was calculated using Cohen’s kappa coefficient (κ = 0.81), indicating strong agreement between reviewers.

### 2.2. Inclusion and Exclusion Criteria

The eligibility criteria were refined and expanded from the original methodology to ensure precise inclusion of relevant studies. Table 3 presents the detailed inclusion and exclusion criteria organized by PICOS elements.

### 2.3. Study Selection Process

**Figure 1:**
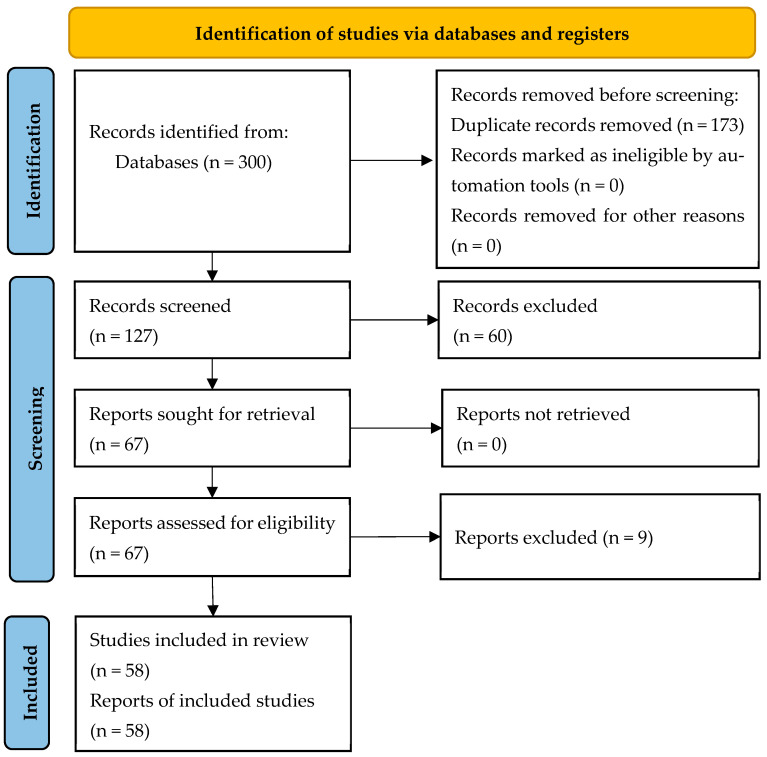
Selection of the relevant papers based on the PRISMA 2020 flow diagram.

The study selection process followed the PRISMA 2020 guidelines and is visually represented in Figure 1, which captures the flow of information through different phases of the review. A total of 300 records were retrieved. After removing duplicates, 127 titles and abstracts were screened. Of these, 67 full-text articles were assessed for eligibility. A final total of 58 articles met the inclusion criteria.

### 2.4. Quality Assessment and Risk of Bias

A methodical quality assessment process was implemented to evaluate the included studies. Given the interdisciplinary nature of the research spanning engineering, computer science, and clinical domains, we developed a custom quality assessment tool that incorporates elements from the following:

- The Joanna Briggs Institute (JBI) Critical Appraisal Tools.
- The Quality Assessment of Diagnostic Accuracy Studies (QUADAS-2).
- Additional technical criteria specific to optical sensing technologies.

Table 4 outlines the quality assessment criteria used to evaluate included studies.

Total quality scores were categorized as follows:

- High quality: 20–24 points.
- Moderate quality: 14–19 points.
- Low quality: <14 points.

We assessed each study, with discrepancies resolved through discussion. No studies were excluded based solely on quality assessment, but quality ratings were considered during data synthesis and interpretation

### 2.5. Characteristics of Included Studies

This review included a total of 58 articles, encompassing a broad spectrum of research approaches and publication years. Table 5 presents the distribution of studies by type. The most common category was Validation studies (*n* = 15), reflecting a strong focus on assessing the accuracy and reliability of optical and sensor-based technologies for gait and obesity analysis. Other prevalent study types included Experimental (*n = 7*), Modeling (*n* = 7), and Pilot studies (*n* = 6), indicating both exploratory and applied research directions. Additional types included system design, anthropometric investigations, and comparative analyses, alongside a few Reviews and Systematic Reviews (*n* = 2 each), which served to contextualize existing findings.

**Figure 2:**
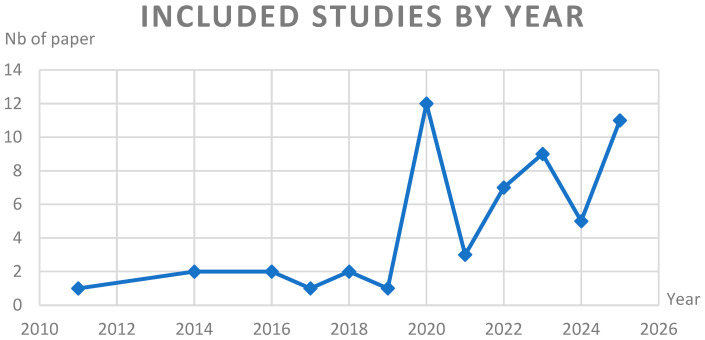
Summary of included studies by year.

Figure 2 below summarizes the temporal distribution of the included studies. The publication dates ranged from 2011 to 2025, with a notable increase in studies over recent years. The peak in publications occurred in 2020 (*n* = 12) and 2025 (*n* = 11), suggesting a growing interest and technological maturation in optical sensing for gait analysis, particularly in obesity-related contexts.

To further illustrate the breadth and methodological diversity, Table 6 provides a subset of studies highlighting the range of technologies used (e.g., depth cameras, motion capture systems, markerless pose estimation with DeepLabCut or OpenPose), validation strategies (e.g., intra-rater reliability, accuracy benchmarks, classification performance), and their specific relevance to obesity. Studies targeting obesity-related gait characteristics represented a significant portion of the included works, aligning with the review’s central research objective.

### 2.6. Chronological Evolution Analysis

**Figure 3:**
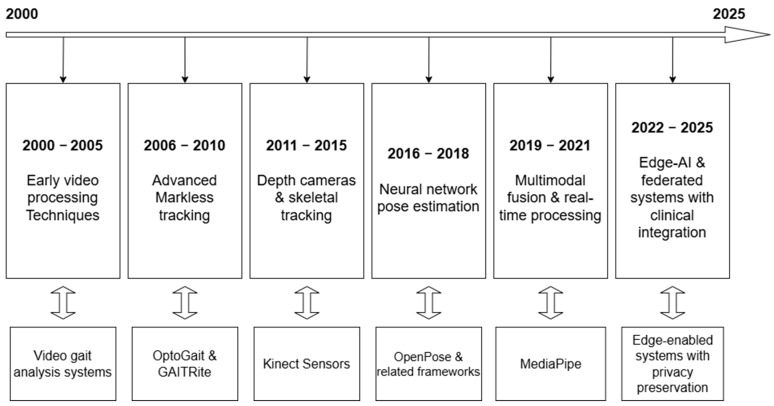
Timeline of key developments in optical sensor technologies for obesity detection.

To capture the technological evolution in the field, we conducted a chronological analysis of optical sensing technologies for obesity detection from 2000 to 2025. Figure 3 illustrates this evolution.

### 2.7. Review Structure

The literature review was organized using a hybrid approach that combines technological taxonomies with application domains, enabling a comprehensive analysis of the field. This structure allows for both technical depth and application relevance.

#### 2.7.1. Primary Organization by Technology

**Optical Gait Analysis Systems**

- Light barrier technologies (e.g., OptoGait);
- Pressure-sensitive walkways (e.g., GAITRite);
- Video-based markerless systems;
- Multi-camera setups.

**Vision-Based Pose Estimation Frameworks**

- 2D pose estimation approaches (e.g., OpenPose, MediaPipe);
- 3D pose reconstruction methods;
- Deep learning architectures (e.g., CNNs, transformers);
- Multi-person tracking systems.

**Depth Sensor-Based Voxel Modeling**

- Structured light systems (e.g., first-generation Kinect);
- Time-of-flight sensors (e.g., Azure Kinect, RealSense);
- 3D body composition analysis;
- Dynamic modeling approaches.

**Hybrid and Multimodal Systems**

- Sensor fusion architectures;
- Combined optical-inertial systems;
- Multi-view integration approaches;
- Ensemble methods.

#### 2.7.2. Secondary Organization by Application Focus

Within each technological category, studies were further organized according to their primary application focus:

**Biomechanical and Kinematic Analysis**

- Spatiotemporal gait parameters;
- Joint angles and ranges of motion;
- Center of mass trajectories;
- Dynamic stability metrics.

**Anthropometric Measurement and Validation**

- Body volume estimation;
- Circumference measurements;
- Body shape analysis;
- Segmental proportions.

**Obesity Classification and Risk Assessment**

- Algorithm development and validation;
- Feature extraction methodologies;
- Classification performance metrics;
- Threshold determination.

**Implementation and Deployment Frameworks**

- Clinical integration pathways;
- Edge computing implementations;
- Privacy-preserving architectures;
- Real-world deployment considerations.

This dual organizational structure enables the identification of both technological trends and application-specific challenges across the field of optical sensor-based obesity detection.

To summarize, his methodology ensures transparency and reproducibility of the literature review process. By adhering to PRISMA, we aim to strengthen the credibility of our findings and align with the best practices required for publication in high-impact journals. Future research would benefit from the establishment of a shared repository of benchmark datasets for cross-validation and method comparison.

## 3. Optical Sensors Technologies for Gait Analysis in Obesity Detection

As obesity has become a global health concern, it is also associated with considerable motor impairments, particularly affecting gait and balance. Objective gait analysis techniques offer valuable tools for assessing these impairments and potentially identifying obesity-related gait alterations.

In this part, we examine the application of optical sensor-based systems for gait analysis in individuals with obesity compared to normal-weight controls, drawing upon insights from the provided literature. We discuss the principles and hardware configurations of these systems, explore the gait biomarkers identified in the context of obesity, and analyze the technical advantages and limitations inherent in their application.

### 3.1. Sensor Technologies Overview/Optical Gait Sensing for Obesity Detection

Gait analysis traditionally relies on either semi-subjective observations or objective measurements using various sensor technologies [7,15]. Objective methods leverage technological advancements to quantify gait parameters with greater accuracy, exactitude, repeatability, and reproducibility compared to subjective assessments [7]. These objective techniques can be broadly categorized based on sensor placement: Non-Wearable Sensors (NWS) and Wearable Sensors (WS) [7]. Motion capture systems primarily fall under the NWS category, requiring controlled laboratory settings where subjects walk along defined walkways equipped with sensors [7].

Gait analysis using sensors in the context of obesity involves two approaches: floor sensor systems and image processing technologies based on video capture. Traditional systems like force platforms and pressure mats have historically served as gold standards for gait analysis, but they are not based on optical principles. This review focuses on vision-based systems and optical timing tools such as OptoGait and video capture methods that leverage photometric sensing.

#### 3.1.1. Optical Timing Systems

While not explicitly detailed as a separate category distinct from other floor or image-based systems in the sources, the description of systems like OptoGait suggests a form of optical timing and measurement [8].

OptoGait is described as a portable photoelectric cell system used for clinical assessment of static and dynamic foot pressures and quantifying spatio-temporal parameters. It works by measuring foot movements and space-temporal relationships using photoelectric cells. The system is noted for its reliability in clinical assessment [8]. While technically leveraging photoelectric principles rather than image processing or traditional force/pressure plates, it functions similarly to some floor-based systems by assessing gait on a walkway and is often used to derive similar spatio-temporal parameters.

#### 3.1.2. Video-Based Capture (Image Processing)

Image processing (IP) techniques utilize cameras to capture and analyze gait [7]. These systems extract essential gait features from images [7]. They range from single-camera systems to more complex multi-camera setups [7,15].

- Marker-Based Systems: These optical motion capture systems track targeted joints and orientations using reflective markers placed on the body [15]. They use multi-camera stereophotogrammetric video systems to compute the 3D localization of these markers, determining joint positions and body segment orientations [15].
- Markerless Systems: These systems use a human body model and image features to determine shape, pose, and joint orientations without the need for markers [15]. Recent work utilizes computer vision techniques and deep neural networks to extract 2D skeletons from images for gait analysis, even exploring privacy-preserving methods by processing encrypted images [9]. Examples include systems based on single cameras, Time of Flight sensors, Stereoscopic Vision, Structured Light, and IR Thermography [7].

Image processing systems allow for individual recognition and segment position analysis [7]. They offer advantages like relatively simple equipment setup for single cameras but can involve complex analysis algorithms and high computational costs for more advanced configurations [7]. They require controlled laboratory environments [15].

In summary, non-wearable systems for gait analysis in the context of obesity primarily involve floor-mounted force/pressure sensors and camera-based image processing systems. Although these systems differ in the specific parameters they measure (forces vs. kinematics) and their technical complexity and cost, they provide objective, quantitative data in a controlled setting [7,15]. The OptoGait system, a photoelectric cell-based system, also falls under this umbrella of fixed-location measurement systems used for gait assessment [8].

### 3.2. Applications in Obesity Context: Identified Biomarkers

Obesity is clearly linked to motor impairments, including deficits in gait and balance [10]. Individuals with obesity exhibit differences in movement and gait compared to those with normal weight, contributing to an increased risk of falls and stumbling [10]. Gait analysis using objective methods, including optical sensor-based systems, is applied to quantify these differences and identify specific gait alterations or biomarkers associated with obesity [5].

The goal is to capture gait and balance impairment in individuals with obese BMI and relate it to specific parameters [10]. While one source mentions findings that did not show significant differences in cadence, gait speed, stride duration, daily step count, or double support time between normal and obese BMI categories, it also notes that these findings diverge from existing literature [5]. Other sources and the research questions themselves highlight the expectation and investigation of such differences [5,10].

Typical gait parameters investigated in the context of obesity using objective systems include spatiotemporal parameters, kinematics, and kinetics [5,10,15].

#### 3.2.1. Spatiotemporal Parameters

These include metrics like gait speed, step length, stride length, cadence, step width, step angle, step time, swing time, stance time, and double support time [5,7]. Studies aim to investigate variances in these parameters between obese and normal weight groups. Koinis et al. suggest that increasing BMI is associated with decreased gait speed and that obesity significantly increases the likelihood of falls [5]. Koinis et al. note that people with obesity may experience up to a 15% reduction in gait speed and a 25% decrease in step length compared to those with normal BMI, although their own study did not find significant differences in some parameters [5]. Spatiotemporal parameters, especially walking speed and step length, are considered clinically important indicators [7].

#### 3.2.2. Kinematics

This describes the movement of joints and body segments, including range of motion and segment acceleration [15,16]. While less explicitly detailed in relation to optical image systems and obesity in the provided excerpts compared to spatiotemporal parameters, biomechanical studies of obesity-related gait do investigate joint mechanics [5,7,10,11]. Image processing systems (marker-based and markerless) are capable of measuring joint angles and segment position/orientation [7,15].

#### 3.2.3. Kinetics

This focuses on the forces and moments that cause movement, such as Ground Reaction Forces (GRF), muscle force, and joint momentum [15,16]. Floor sensor systems, particularly force platforms, are designed to measure GRF [7,15]. These kinetic parameters provide insight into the biomechanical effects of increased body mass on the musculoskeletal system during gait [5,7,11].

In addition to these quantitative parameters, gait analysis can also reveal qualitative aspects and patterns, such as gait symmetry and postural balance [7,11,15]. While specific findings on gait asymmetry directly measured by optical sensors in obese individuals are not detailed across the sources, a study on overweight and obese children mentions assessing pelvic symmetry indices using a wearable system (BTS G-WALK, which uses inertial sensors) [11]. Postural balance is a key problem associated with conditions affecting gait, including obesity [7,10,15]. Gait and balance analysis are crucial for understanding locomotor and functional impairments [15].

Therefore, motion capture systems, particularly those using camera-based optical sensors, are used to objectively measure spatiotemporal, kinematic, and kinetic parameters that serve as biomarkers of obesity-related gait impairments, including potential changes in speed, step/stride length, timing, forces, joint movements, and overall gait pattern and stability [5,7,10,15].

In summary, gait biomechanics in individuals with obesity are characterized by systematic and reproducible deviations spanning spatiotemporal, kinematic, and kinetic parameters. These alterations are indicative of compensatory mechanisms employed to preserve stability and locomotor efficiency in the context of increased body mass. Importantly, such biomechanical adaptations are associated with heightened musculoskeletal loading, elevated risk for injury, and diminished functional mobility. Table 7 provides a comprehensive synthesis of the principal gait features consistently identified in individuals with obesity, highlighting obesity-specific deviations and their potential clinical ramifications.

### 3.3. Technical Advantages and Limitations

Objective gait analysis systems, including those based on optical sensors, offer significant advantages over traditional semi-subjective methods by providing accurate and quantitative data [7]. However, they also present technical limitations, particularly when considering their application in diverse settings and populations, such as individuals with obesity.

#### 3.3.1. Precision vs. Portability Trade-Offs 

Non-wearable sensor (NWS) systems, such as ground reaction force (GRF) platforms, pressure sensor mats, and optical timing systems like OptoGait, are widely recognized as the gold standard for gait measurement in controlled laboratory environments due to their high accuracy and repeatability [15]. For instance, GRF plates offer high accuracy with minimal load error, and pressure sensor mats can achieve high recognition rates [1]. These systems enable the simultaneous measurement of multiple gait parameters with minimal error, particularly in detecting spatial and temporal gait characteristics. However, this precision comes at the expense of portability. NWS setups are typically bulky, require specialized facilities and calibration procedures, and are impractical for use in real-world or ambulatory settings [7,14].

In contrast, wearable sensor (WS) systems offer greater portability and the capacity to monitor gait over extended periods in natural environments. Although not the primary focus of this review, WS systems—such as those relying on inertial measurement units—are increasingly used for ecological gait tracking [17]. Nonetheless, they often exhibit lower accuracy and reliability compared to NWS systems, especially in capturing fine-grained kinematic or kinetic data [7,14].

A third and increasingly prominent category includes optical video-based systems, such as those using RGB or depth cameras (e.g., Microsoft Kinect, Intel RealSense), which provide markerless motion capture capabilities [16]. These systems leverage computer vision and depth sensing to extract joint positions and spatiotemporal features. While they offer a non-invasive and cost-effective alternative for gait analysis, their performance can be affected by environmental factors, such as lighting, clothing variability, and, critically, occlusion—particularly in individuals with higher BMI, where body segments may obscure each other or deform expected anatomical contours.

#### 3.3.2. Environmental Dependencies, Calibration Needs, and Other Factors

Optical sensor-based systems can be sensitive to environmental factors and require careful setup and calibration.

- Controlled Environment: Optical NWS requires controlled research facilities. Subjects must walk on a clearly marked walkway [7].
- Calibration: Both optical sensors and camera systems require calibration. For instance, stereoscopic vision systems involve complex calibration, and structured light systems also require calibration [7]. While the sources do not detail the specific calibration requirements for obese subjects, increased body size or altered gait patterns could potentially influence calibration procedures or accuracy.
- Subject-Specific Variance: While not unique to optical systems, individual variations in gait patterns are inherent. In the context of obesity, larger body mass significantly affects biomechanics and gait patterns [5,7,11]. Accurately capturing these subject-specific variations requires robust measurement techniques. Image processing systems that track body segments or skeletons may need to account for differences in body shape and soft tissue movement in obese individuals [9].

#### 3.3.3. Limitations Specific to Optical Sensor-Based Gait Analysis Systems

Single-camera systems have simple equipment but require complex analysis algorithms. Stereoscopic vision has complex calibration and high computational cost. Time of flight systems can have problems with reflective surfaces. IR Thermography requires considering emissivity, absorptivity, reflectivity, and transmissivity of materials. Extracting parameters like step length from image-based systems can sometimes be more accurate than methods used in some WS systems.

Despite their recognized precision, the practical deployment of optical sensor-based gait systems remains constrained by inherent technical and operational limitations [7,15]. These systems typically require controlled conditions and are susceptible to calibration drift, occlusion artifacts, body shape specificity, and environmental variability [7,15,18]. In populations with obesity, additional challenges arise due to body morphology, which can obstruct key anatomical landmarks and disrupt algorithmic tracking. Moreover, the static setup of such systems restricts their use to isolated assessments rather than continuous, real-world monitoring. As a result, while these technologies provide valuable biomechanical insights, their current configuration limits scalability and generalizability especially in the context of heterogeneous and high-BMI populations where adaptability and robustness are essential [5,7,11]. 

To contextualize these challenges, Table 8 provides a detailed comparative synthesis of current optical sensor technologies, highlighting their core principles, accuracy profiles, sensitivity metrics, and obesity-specific applications, as well as their respective operational advantages and limitations across marker-based, markerless, depth, and photoelectric systems.

### 3.4. Analytical Models for Human Motion Capture, Gait Analysis, and Obesity Detection

The scholarly literature on human motion capture, gait analysis, and obesity detection encompasses a diverse array of analytical methodologies. These range from classical statistical frameworks and time-series models to sophisticated deep learning architectures, each harnessing distinct sensor modalities and data representations to address complex biomechanical and physiological inference tasks.

#### 3.4.1. 1. Time-Series Analytical Frameworks

Time-series analysis is a fundamental approach for processing sequential data, such as sensor readings over time. In the context of human movement, gait data collected from sensors are inherently time-series in nature

*Feature Extraction*

Raw sensor signals are segmented via sliding window techniques (e.g., Gaussian and Box filters) [27]. From these segments, a suite of statistical descriptors—mean, standard deviation, variance, skewness, kurtosis, root mean square error, autocorrelation, and autocovariance—are systematically derived. This process facilitates dimensionality reduction while preserving critical temporal dynamics and attenuating noise [27].

*Applications*

The extracted features serve as inputs to conventional classifiers for predictive tasks such as Body Mass Index (BMI) estimation and age group categorization. The influence of physiological traits on gait patterns enables their inference through sequential data analysis [27].

#### 3.4.2. 2. Deep Learning with Convolutional Neural Networks (CNNs)

CNNs have emerged as a principal tool for hierarchical feature extraction from both spatial and temporal data, with broad application in human motion analysis and obesity detection [6,31].

*Human Pose Estimation and Motion Capture*

- Markerless Motion Capture

CNNs are central to markerless motion capture, which estimates human pose from images or videos without physical markers [12,19,31]. Advanced frameworks such as DeepLabCut employ deep residual networks (e.g., ResNet-50) for precise localization of anatomical landmarks in video frames [12]. Similarly, DeeperCut, leveraging fully convolutional ResNet architectures, enhances multi-part detection robustness through expanded receptive fields [32,33]. OpenPose is another deep learning-based method for 2D pose estimation using part affinity fields [19,34,35].

- 3D Human Reconstruction

CNNs are applied in multi-view and volumetric contexts to reconstruct high-fidelity 3D human models, utilizing voxel-based super-resolution and implicit 3D representation learning to improve geometric accuracy [22,24,35].

*Obesity Detection*

- Thermal Imaging

Both custom and pre-trained CNNs (e.g., VGG16, ResNet, DenseNet) are deployed to classify thermal images of anatomical regions such as the abdomen, forearm, and shank, discriminating between obese and non-obese phenotypes by identifying patterns associated with brown adipose tissue activity [31].

- Gait Analysis with Smartphone Sensors

Although it is not in the scope of this review, it is important to mention that one-dimensional CNNs (1D CNNs) are specifically designed to handle 1D signals like those from smartphone accelerometers and gyroscopes to classify individuals as normal or overweight/obese based on distinctive gait signatures [6].

- Abnormal Gait Detection

CNNs are also utilized to distinguish between normal and pathological gait by analyzing 2D skeletal representations extracted from video sequences [9].

*Architectural Variants*

Convolutional neural network (CNN) architectures in gait and obesity analysis have adapted to various data types, with structural differences reflecting specific input requirements such as time-series, volumetric, or graph-based data. Table 9 outlines key architectural variants from the literature, emphasizing their input domains and targeted applications.

#### 3.4.3. Autoencoders and Generative Modeling

While explicit references to Variational Autoencoders (VAEs) are limited, the literature details a variety of generative modeling strategies for 3D reconstruction and latent feature representation.

- 3D Shape Reconstruction:

Generative models synthesize 3D human body shapes from sparse 2D observations, employing deformable templates, mesh autoencoders, and adversarial networks to produce individualized geometric and textural reconstructions [24,35].

- Abnormal Gait Analysis:

Long Short-Term Memory (LSTM) autoencoders are implemented for anomaly detection, identifying deviations from normative gait patterns in daily activities [9].

#### 3.4.4. Traditional and Hybrid Analytical Approaches

*Traditional Classifiers*

Traditional machine learning classifiers are still prevalent in gait and obesity research, especially with manually extracted features from time-series or image data. These models offer interpretability, low computational cost, and reliable performance on structured tasks. Table 10 below lists the main traditional classifiers reported in the reviewed studies.

These models are routinely employed for both classification and regression, including the integration of multimodal sensor data.

*Hybrid Models*

Hybrid models combine different learning paradigms, typically using convolutional layers for spatial features and recurrent layers for temporal patterns. These architectures are well-suited for the complex, sequential, and multi-modal data often found in gait and obesity research.

- CNN–LSTM Architectures:

These combine the spatial feature extraction capabilities of CNNs with the temporal modeling strengths of LSTMs, offering enhanced performance for sequential gait data in obesity identification [6].

- RNN–CNN Networks:

Hybrid architectures that integrate recurrent and convolutional layers are utilized for abnormal gait detection, leveraging multimodal data such as 3D skeletal trajectories and plantar pressure distributions [9].

#### 3.4.5. Statistical Modeling and Validation Techniques

Statistical methodologies complement machine learning by enabling parameter estimation, population-level shape modeling, and rigorous system validation.

*Shape Modeling and Anthropometry*

Statistical models capture and quantify 3D human shape variation across populations, supporting the estimation of anthropometric parameters [36].

*Dimensionality Reduction*

Principal Component Analysis (PCA) is extensively employed to characterize variability in 3D body shapes and facilitate body measurement prediction [36].

*Validation Techniques*

Studies utilize statistical tests including *t*-tests, ANOVA, Pearson’s correlation coefficient (PCC), intraclass correlation coefficient (ICC), and Bland–Altman analysis, to assess measurement agreement and validate system performance [8,12,19,34].

In this section, we highlighted the critical role of objective gait analysis in understanding and quantifying the motor impairments associated with obesity. Optical sensor systems, floor sensor technologies (force platforms, pressure systems) and video-based capture (marker-based and markerless image processing), represent key non-wearable sensor (NWS) approaches used in this field [7,15]. These systems are capable of measuring important gait biomarkers such as spatiotemporal parameters, kinematics, and kinetics, which are known to be altered by increased body mass and contribute to mobility issues and fall risk in individuals with obesity [5,7].

While NWS optical systems offer high accuracy and the ability to collect comprehensive data in controlled settings, they are limited by their lack of portability, high cost, need for specialized expertise, and susceptibility to environmental and subject-specific factors [7,15,18]. Despite these limitations, they remain valuable tools for detailed clinical and research assessments of gait mechanics in obesity.

We also presented the evolution of analytical models in human motion capture, gait analysis, and obesity detection that reflects a clear methodological trajectory: from traditional, hand engineered approaches to sophisticated, data-driven deep learning architectures capable of end-to-end feature learning. The emergence of hybrid models underscores a paradigm shift, leveraging the complementary strengths of multiple analytical frameworks to improve interpretability, accuracy, and generalizability in biomechanical and obesity-related research.

Future advancements, particularly in areas like miniaturization, power efficiency, and sophisticated algorithms, are focused on improving wearable technologies to potentially bridge the gap in measurement capacity and accuracy with NWS, enabling long-term, real-world gait monitoring. However, for detailed, high-precision laboratory-based analysis, optical sensor systems continue to play a significant role in uncovering the complex interplay between obesity and gait dynamics. Further research utilizing these objective techniques is essential for refining our understanding of obesity-related gait abnormalities and developing targeted interventions.

## 4. Markerless Video-Based Pose Estimation Technologies

Markerless pose estimation represents a revolutionary approach to human motion analysis, enabling the extraction of kinematic data without the need for physical markers attached to subjects. This technology has seen rapid advancement in recent years, primarily driven by developments in computer vision and deep learning. Unlike traditional marker-based motion capture systems that require specialized hardware and controlled laboratory environments, markerless systems operate with standard cameras in diverse settings, making them accessible for widespread applications in healthcare, sports science, biomechanics research, and human–computer interaction. This part of the review examines current markerless video-based pose estimation technologies, focusing on algorithms, validation, challenges related to body morphology diversity, and advancements in hybrid sensing approaches.

### 4.1. Key Algorithms and Platforms

#### 4.1.1. OpenPose

OpenPose represents one of the pioneering deep learning-based frameworks for real-time multi-person human pose detection. Developed at Carnegie Mellon University, it enabled the simultaneous detection of multiple individuals within a single image or video frame. OpenPose employs a bottom-up approach that first detects body parts across the entire image and then associates them to form complete human skeletons.

The architecture of OpenPose is built upon a multi-stage convolutional neural network (CNN) that processes images through two main branches: one for body part detection and another for part association. This two-branch approach enables the system to maintain high accuracy even when multiple people appear in the scene with overlapping body parts. The network generates confidence maps for each body part location and part affinity fields (PAFs) that encode the degree of association between parts, allowing the system to determine which body parts belong to the same person.

OpenPose can jointly detect human body, foot, hand, and facial keypoints, providing a comprehensive representation of human pose. The standard model identifies 25 body keypoints, including major joints like shoulders, elbows, wrists, hips, knees, and ankles, as well as facial landmarks. Extended models incorporate additional keypoints for hands and detailed facial features, resulting in a total of 135 keypoints per person when using the full model.

The versatility of OpenPose has led to its application across diverse domains. In biomechanics research, it has enabled the analysis of sports performance without interfering with athletes’ natural movements. In 2020, Nakano et al. developed a 3D markerless motion capture technique using OpenPose with multiple synchronized cameras to evaluate motor performance tasks including walking, jumping, and ball throwing [14]. They found that approximately 47% of measurements had mean absolute errors below 20mm compared to marker-based systems, with 80% below 30mm [14].

#### 4.1.2. MediaPipe

MediaPipe Pose is another significant deep learning-based framework for human pose estimation, developed by Google. Unlike OpenPose’s bottom-up approach, MediaPipe typically employs a top-down methodology that first detects persons in the image and then estimates the pose for each detected individual. This approach generally works well when the number of people in the scene is limited, making it particularly suitable for applications focusing on a single subject or a few individuals.

MediaPipe Pose Estimation is based on the BlazePose architecture, which was specifically designed for real-time performance on mobile devices [37]. The system provides 33 3D keypoints in real-time, representing a superset of the 17 keypoints from the COCO dataset (commonly used in many pose estimation systems). These additional points provide more detailed tracking of the face, hands, and feet, enhancing the granularity of pose information [37]. The pipeline of MediaPipe Pose first detects a person in the image using a face detector and then predicts the keypoints, assuming that the face is always visible [37].

A distinctive feature of MediaPipe is its optimized performance for mobile deployment. On devices like the Samsung Galaxy S23 Ultra with the Snapdragon 8 Gen 2 chipset, the inference time can be as low as 0.826 ms, with a peak memory range of 0–1 MB [26]. This exceptional efficiency makes MediaPipe an excellent choice for real-time applications on edge devices where computational resources are limited.

MediaPipe Pose is primarily designed for fitness applications involving a single person or a few people in the scene [37]. Its applications include yoga pose correction, fitness tracking, physical therapy, and gesture-based interfaces. The framework is easily accessible through Python 3.7+ packages and can be configured to run on cloud-hosted devices using platforms like the Qualcomm AI Hub [26].

#### 4.1.3. DeepLabCut

DeepLabCut represents a different approach to pose estimation, originally developed for markerless tracking of animals in research settings. Created by Mathis et al., DeepLabCut leverages transfer learning to achieve high-performance pose estimation with relatively small training datasets, making it particularly valuable for specialized applications where large annotated datasets may not be available [33].

The architecture of DeepLabCut was initially inspired by DeeperCut, a state-of-the-art algorithm for human pose estimation by Insafutdinov et al. [32], which inspired the name for the toolbox. However, since its inception, DeepLabCut has evolved substantially, incorporating various backbone networks including ResNets, MobileNetV2, EfficientNets, and the custom DLCRNet backbones. This flexibility in network architecture allows users to balance accuracy and computational efficiency based on their specific requirements.

A key strength of DeepLabCut is its ability to achieve high accuracy with limited training data, typically requiring only a few hundred labeled frames to generate reliable pose estimates for novel videos [33]. This is achieved through transfer learning, where pre-trained networks (typically trained on ImageNet) are fine-tuned for specific pose estimation tasks. The developers have demonstrated that this approach works effectively across species including mice, flies, humans, fish, and horses.

In addition to its 2D pose estimation capabilities, DeepLabCut also supports 3D pose reconstruction using multiple cameras or even from a single camera with appropriate training data [38]. The framework has been extended to support real-time processing through DLClive, enabling applications that require immediate feedback based on pose information [38].

While DeepLabCut was originally developed for animal tracking, its principles and approaches have been successfully applied to human subjects as well [39]. The framework is particularly valuable in research contexts where custom keypoint definitions may be needed, or where the specifics of the application differ from the standard human pose estimation use cases [12].

The table below compares three popular pose estimation algorithms—MediaPipe, OpenPose, and DeepLabCut—based on reported metrics (RMSE, PCK) and key system features such as keypoint coverage, efficiency, and limitations. This summary highlights each algorithm’s practical strengths and weaknesses for motion capture applications requiring different levels of accuracy, speed, and adaptability.

Below in Table 11, is a comparative summary outlining the performance characteristics of the reviewed pose estimation algorithms: MediaPipe, OpenPose, and DeepLabCut.

### 4.2. Validation and Accuracy

#### 4.2.1. Comparison with Gold Standard Systems

The validation of markerless pose estimation systems against gold standard marker-based motion capture is essential for establishing their reliability in scientific and clinical applications. Optical marker-based systems, such as Vicon or OptiTrack, remain the reference standard in biomechanics research due to their sub-millimeter accuracy in controlled environments.

A comprehensive evaluation of OpenPose-based markerless motion capture was conducted by Nakano et al., comparing it with optical marker-based systems during various motor tasks including walking, countermovement jumping, and ball throwing [14]. The study employed multiple synchronized cameras to reconstruct 3D poses from OpenPose’s 2D estimates and compared the resulting joint positions with those measured by a marker-based system. The differences were quantified using mean absolute error (MAE) between corresponding joint positions [14].

The results summarized in Table 12 revealed that approximately 47% of all calculated mean absolute errors were below 20 mm, and 80% were below 30 mm, indicating reasonable accuracy for many applications [14]. However, approximately 10% of errors exceeded 40 mm, primarily due to failures in OpenPose’s 2D tracking, such as incorrectly recognizing objects as body segments or confusing one body segment with another [14]. These findings suggest that while markerless systems can approach the accuracy of marker-based systems for many applications, they still face challenges in robustly tracking all body segments across diverse movements and viewing conditions [13].

The accuracy of markerless systems varies considerably across different joints and movement types. Generally, larger and more visible joints such as the shoulders, hips, and knees tend to be tracked more reliably than smaller joints like the wrists, ankles, and fingers. Additionally, movements that involve rapid motion, occlusion, or unusual poses can challenge the performance of current algorithms, leading to increased error rates [19].

It is important to note that while absolute position accuracy may still lag behind marker-based systems, many applications primarily require accurate relative motion patterns or joint angles, which markerless systems can often provide with sufficient reliability. This makes them viable alternatives for applications where the convenience of markerless tracking outweighs the need for the highest possible accuracy.

#### 4.2.2. Comparison with IMU Systems

IMUs excel at capturing segment orientations and can operate without line-of-sight constraints, making them suitable for complex environments with occlusions. However, they struggle with position drift over time and require careful calibration. Markerless video systems, in contrast, can provide absolute position information without drift but require continuous visibility of body segments to the cameras.

The complementary nature of these technologies has led to increasing interest in hybrid systems that combine IMUs with video-based tracking to leverage the strengths of each approach. Such systems can use visual information to correct IMU drift while using IMU data to fill gaps during visual occlusions.

#### 4.2.3. Body Morphology Effects on Detection

The impact of body morphology, particularly body mass index (BMI), on the accuracy of markerless pose estimation represents a significant challenge for these technologies. High BMI can affect pose estimation accuracy through several mechanisms. First, increased adipose tissue can change the visual appearance of joints, making their precise localization more difficult. Second, in individuals with higher BMI, certain joints may be partially occluded by soft tissue, reducing their visibility to the camera. Third, the standard body proportions assumed by many pose estimation algorithms may not accurately represent individuals with higher BMI, potentially leading to systematic errors in keypoint placement.

Limited research has directly quantified these effects, but clinical experience and preliminary studies suggest that pose estimation accuracy generally decreases as BMI increases, particularly for joints of the lower extremities. This creates a significant challenge for applications in healthcare settings, where individuals with higher BMI may be precisely those who would benefit most from motion analysis for conditions like osteoarthritis, genu valgum, diabetic gait disorders, or rehabilitation monitoring.

To address these challenges, several approaches have been proposed. One approach involves creating more diverse training datasets that include individuals across the full spectrum of body sizes and shapes. Another approach uses adaptive algorithms that can adjust their keypoint detection strategies based on the detected body morphology. Some researchers have also explored the use of additional sensors, such as depth cameras, to provide supplementary information that can improve joint localization in challenging cases.

The ability to accurately track movements across diverse body morphologies remains an important frontier for markerless pose estimation research, with implications for the equity and inclusivity of these technologies in healthcare and other domains.

### 4.3. Obesity-Related Gait Signatures

#### 4.3.1. Technical Challenges

Markerless pose estimation faces several technical challenges when applied to individuals with obesity, particularly in the context of gait analysis. The first major challenge is joint occlusion, which occurs when adipose tissue or limb positioning prevents clear visual access to joint centers. This is especially problematic for the hip joints, which may be obscured by abdominal or thigh tissue, and for the knees, which can be partially hidden during certain phases of the gait cycle.

Over-segmentation represents another challenge, where the algorithm incorrectly identifies multiple keypoints where only one should exist. This can occur when the visual appearance of body segments in high-BMI individuals differs significantly from the training data used to develop the pose estimation model. For example, the algorithm might mistakenly identify multiple knee joints due to the different contour of the leg in individuals with higher BMI.

Signal processing adaptations have been developed to address these challenges. These include temporal filtering approaches that maintain continuity of joint trajectories based on biomechanical constraints, preventing physically impossible jumps in joint positions between frames. Some systems also incorporate anatomical constraints and body-specific calibration procedures to adapt their models to individual body morphologies.

Multi-view approaches can significantly mitigate occlusion issues by providing alternative angles from which to observe partially hidden joints. When a joint is occluded from one camera’s perspective, it may be visible from another, allowing the system to maintain tracking. Advanced systems can dynamically weight the confidence of detections from different cameras based on their viewing angle relative to each body segment.

Addressing these technical challenges is essential for developing inclusive motion analysis technologies that can serve diverse populations. The most promising approaches combine algorithmic improvements with hardware solutions like strategic camera placement to maximize visibility of key anatomical landmarks.

#### 4.3.2. Biomechanical Alterations

Obesity is associated with several characteristic alterations in gait biomechanics that pose estimation systems must accurately capture to provide clinically relevant information. Understanding these patterns is essential both for developing more robust tracking algorithms and for interpreting the resulting kinematic data in clinical contexts.

Altered joint angle trajectories represent one of the most significant gait modifications in individuals with obesity. Typically, these include reduced knee flexion during swing phase, decreased hip extension during late stance, and modified ankle kinematics throughout the gait cycle. These alterations are believed to result from a combination of increased joint loading, altered muscle function, and adaptations to maintain stability with changed body mass distribution.

Increased trunk lean is another common characteristic of gait in individuals with higher BMI. This forward inclination of the trunk shifts the center of mass anteriorly, potentially reducing the muscular effort required to initiate forward progression during walking. Accurately quantifying trunk lean is important for assessing energy expenditure during gait and for understanding compensatory mechanisms that may increase risk for back pain or other musculoskeletal issues.

Lateral sway patterns also differ in individuals with obesity, with typically increased mediolateral center of mass displacement during walking. This increased lateral movement requires additional stabilizing mechanisms and may contribute to higher energy costs of walking. Capturing these subtle movements requires pose estimation systems with high accuracy in tracking the relative positions of the pelvis, lower extremities, and trunk.

Markerless systems must be capable of accurately measuring these biomechanical alterations to provide clinically meaningful assessments. Validation studies specifically examining the accuracy of these systems in capturing obesity-related gait signatures are limited but represent an important area for future research.

#### 4.3.3. Clinical Applications

Despite the challenges, markerless pose estimation offers significant potential for clinical applications related to obesity and associated movement disorders. The non-invasive nature of these systems makes them particularly valuable for longitudinal monitoring, where repeated assessments are needed to track changes over time.

In weight management programs, objective quantification of gait parameters can provide valuable feedback on the functional improvements resulting from weight loss. Parameters such as step length, walking speed, joint ranges of motion, and stability measures can demonstrate functional gains that may motivate continued adherence to intervention programs. Markerless systems enable these measurements to be taken in clinical settings without the time-consuming application of markers or specialized equipment.

For surgical interventions such as bariatric surgery or joint replacements, markerless motion analysis can help document functional outcomes and guide rehabilitation strategies. The ability to conduct these assessments quickly and easily facilitates their integration into routine clinical care, rather than being limited to specialized research settings.

Telehealth applications represent another promising domain, where markerless systems using standard webcams could enable remote assessment of movement function. This could be particularly valuable for monitoring patients in rural or underserved areas where access to specialized gait laboratories is limited.

As these technologies continue to improve in accuracy and robustness across diverse body morphologies, their integration into standard clinical care pathways for obesity and related conditions becomes increasingly feasible, potentially transforming the assessment and management of movement-related complications.

### 4.4. Depth and Hybrid Systems

#### 4.4.1. RGB-D Framework

RGB-D systems combine traditional color images (RGB) with depth information (D), creating a more comprehensive representation of the 3D scene. While standard RGB cameras capture only the visual appearance of subjects, depth sensors provide direct measurements of the distance between the sensor and each point in the scene. This additional dimension of information can significantly enhance the accuracy and robustness of pose estimation, particularly in challenging scenarios involving occlusions or unusual body positions.

The Microsoft Kinect V2 represents one of the most widely used RGB-D platforms for human motion capture. It combines a standard RGB camera with an infrared time-of-flight depth sensor that provides pixel-wise distance measurements. The integration of depth data allows the system to disambiguate between overlapping body parts and more accurately localize joints in 3D space, even when their appearance in the RGB image alone might be ambiguous.

The processing pipeline for RGB-D pose estimation typically involves several stages. First, the depth information is used to segment the human figure from the background. Next, the segmented depth map is processed to identify body parts using techniques such as random decision forests or deep learning. Finally, a skeletal model is fitted to these detected body parts, considering both the RGB appearance and the 3D structure provided by the depth data.

More recent approaches have incorporated deep learning methods that can jointly process RGB and depth information. These networks are trained to leverage complementary cues from both modalities: appearance features from RGB images and structural information from depth maps. This fusion of information sources has proven particularly effective for robust pose estimation in complex real-world environments.

#### 4.4.2. Accuracy Improvements

The incorporation of depth information provides several significant accuracy improvements for pose estimation, especially in challenging scenarios. First, depth data helps resolve ambiguities in the RGB image by providing direct 3D information about the spatial arrangement of body parts. This is particularly valuable when body parts overlap from the camera’s perspective, which can confuse RGB-only systems.

Second, depth sensors are generally less sensitive to lighting variations than RGB cameras, making them more robust for applications in environments with inconsistent or poor lighting. While strong infrared interference can affect depth sensors, they generally provide more stable measurements across varying ambient light conditions than color-based approaches alone.

Third, depth information facilitates more accurate background segmentation, helping to isolate the human figure from complex environments. This is especially valuable in cluttered scenes where color-based segmentation might struggle to distinguish between the subject and visually similar background elements.

Quantitative studies have demonstrated these advantages, with RGB-D systems typically showing reduced average joint position errors compared to RGB-only approaches when evaluated against marker-based ground truth. The magnitude of improvement varies by joint, with the greatest benefits often seen for joints that are frequently occluded or that lack distinctive color features.

However, it’s important to note that depth sensors have their own limitations, including more restricted range, higher power consumption, and typically lower resolution than RGB cameras. These considerations are particularly relevant for mobile or wearable applications where power and computational resources may be constrained.

#### 4.4.3. Real-World Applications

RGB-D systems have found applications across numerous domains where robust pose estimation in uncontrolled environments is required. In clinical settings, these systems enable functional movement assessment without the need for markers, facilitating the integration of motion analysis into routine care. Applications include gait assessment, balance evaluation, and rehabilitation monitoring, where the system can provide immediate feedback on movement quality and progress.

Home monitoring represents another growing application area, where RGB-D sensors can track movements over extended periods in naturalistic environments. This enables longer-term assessment of mobility patterns and functional status, which may be more representative of real-world capabilities than brief assessments in clinical settings. Privacy concerns in home monitoring can be mitigated by processing data locally and extracting only anonymous skeletal data rather than storing raw RGB images.

Public space analysis for ergonomics, safety, and accessibility represents a third application domain. Here, RGB-D systems can analyze how diverse individuals interact with built environments without requiring individual consent for marker placement. This supports the development of more inclusive design standards that accommodate the full range of human body sizes and movement capabilities.

The continued miniaturization and cost reduction of depth sensing technologies promises to further expand these applications. Emerging systems incorporate depth sensing directly into mobile devices or wearable cameras, enabling pose estimation in increasingly diverse and dynamic environments while maintaining user privacy through on-device processing of sensitive data.

Markerless video-based pose estimation technologies have advanced rapidly in recent years, driven by breakthroughs in deep learning and computer vision. Systems like OpenPose, MediaPipe, and DeepLabCut provide accessible frameworks for human motion analysis across diverse applications, from clinical assessment to sports performance and human–computer interaction.

Validation studies against gold standard marker-based systems indicate that markerless approaches can achieve reasonable accuracy for many applications, with the majority of joint position errors falling below 30 mm in controlled conditions. However, challenges remain in tracking rapid movements, handling occlusions, and accurately capturing the movements of individuals whose body morphologies differ significantly from those represented in training datasets.

The impact of body morphology, particularly higher BMI, on pose estimation accuracy remains an important consideration for clinical applications. Technical challenges including joint occlusion and over-segmentation can affect the reliable tracking of obesity-related gait signatures such as altered joint trajectories, increased trunk lean, and modified lateral sway patterns. Addressing these challenges requires both algorithmic improvements and hardware solutions.

The integration of depth sensing with RGB cameras in hybrid systems offers promising improvements in robustness and accuracy, particularly in complex real-world environments. These RGB-D systems provide complementary information that enhances joint localization, improves robustness to lighting variations, and facilitates better segmentation of human figures from cluttered backgrounds.

Looking forward, the continued development of markerless pose estimation technologies promises to democratize access to human movement analysis, enabling applications that were previously confined to specialized laboratories to be deployed in clinical settings, homes, and public spaces. This expanded access has the potential to transform our understanding of human movement across diverse populations and environments, ultimately contributing to improved healthcare, enhanced performance, and more inclusive design of physical spaces and interfaces.

## 5. Human Voxel Modeling and Anthropometric Estimation

The increasing availability of consumer-grade depth sensors has sparked significant research interest in 3D human body modeling and measurement extraction. This field intersects computer vision, machine learning, and anthropometry to develop methods for accurate body shape reconstruction and measurement estimation. This section examines the current state of research in voxel-based 3D human body modeling, with a focus on anthropometric applications, reconstruction pipelines, and real-world limitations.

### 5.1. Three-Dimensional Body Reconstruction Using Depth Sensors

#### 5.1.1. Depth Sensing Technologies

The evolution of consumer-grade depth cameras has revolutionized 3D human body reconstruction. Time-of-Flight (ToF) cameras, such as Microsoft Kinect V2, measure depth via infrared pulse timing, while stereoscopic cameras like Intel RealSense D435 estimate depth using image parallax. A comparative study by Chuang-Yuan et al. [29] found ToF sensors generally more accurate, with Kinect V2 outperforming RealSense D435 in KinectFusion-based reconstruction.

Microsoft Kinect remains widely used in low-cost 3D scanning due to its accessibility and accuracy. Weiss et al. [40] demonstrated that combining coarse depth data and low-resolution silhouettes from monocular Kinect views enabled accurate 3D modeling, rivaling costly commercial scanners.

#### 5.1.2. Voxel-Based Representation: Principles, Algorithms, and Metrics

Voxel-based representations discretize 3D space into grid cells (voxels), each containing occupancy data. These grids form the foundation of many depth-sensor-based body modeling systems. Li et al. [22] introduced a hierarchical approach that combines coarse 3D reconstruction using Pixel-aligned Implicit Functions (MF-PIFu) with voxel super-resolution (VSR) using multi-stage 3D convolutional neural networks. This significantly improved geometric accuracy.

Chuang-Yuan et al. [29] showed that increasing voxel resolution in KinectFusion from 128 to 512 voxels/m yielded diminishing returns beyond 256 voxels/m, indicating an optimal trade-off between detail and processing cost. Metrics like volumetric RMSE, WHR estimation accuracy, and surface roughness are used to assess performance.

Table 13 summarizes the methodological principles, algorithms, and performance evaluations of voxel-based and 3D shape reconstruction approaches discussed above, including recent developments like VSR, SPLATNet, and model-based anthropometry.

#### 5.1.3. Single-View vs. Multi-View Reconstruction

Multi-view reconstruction remains the gold standard for completeness and surface fidelity. Li et al. [22] demonstrated that combining multiple views yields accurate 3D models with fewer occlusions using memory-efficient implicit functions.

However, practical constraints have driven improvements in single-view approaches. For instance, Pixel2Pose [43] uses high-resolution ToF and intensity images from Kinect, trained with supervised learning to estimate 3D poses of multiple subjects from single-view input. Though spatial resolution is lower, ToF data compensates by capturing temporally rich signals.

#### 5.1.4. Statistical Parametric Body Models (SCAPE and SMPL)

Standard parametric models offer compact, interpretable representations of body shape and pose. The SCAPE (Shape Completion and Animation for PEople) model [40] distinguishes pose from shape by learning from 3D scans, enabling consistent body shape estimation across multiple partial views. It is robust to occlusions, scan noise, and pose variability, and can reconstruct hidden body regions by applying learned priors.

Complementing this, the SMPL (Skinned Multi-Person Linear) model offers a differentiable, low-dimensional representation suitable for deep learning integration. It decouples shape (β ∈ ℝ^10^) and pose (θ) using learned blend shapes, supporting high-resolution reconstruction from 2D or 3D features. Though powerful, SMPL’s limited shape parameters and focus on unclothed meshes constrain its application in clinical settings without preprocessing.

Both models facilitate measurement prediction, pose estimation, and shape completion, and serve as intermediaries between raw data and clinically relevant anthropometric features [36].

### 5.2. Applications in Body Composition Analysis

#### 5.2.1. Anthropometric Measurement Extraction

Voxel-based reconstructions support automated extraction of measurements like limb lengths and body circumferences. Tsoli et al. [36] showed that fitting a deformable 3D model to scans improves measurement accuracy, particularly when aggregating scans from multiple poses. Alexa’s study at Philips [28] used perimeter-based features (waist, thigh, neck) to predict fat percentage with RMSE = 2.22% in pregnant women.

#### 5.2.2. Waist-to-Hip Ratio and Volumetric Indices

The waist-to-hip ratio (WHR) represents a powerful predictor of health risks associated with fat distribution. LeanScreen technology [42] calculates it using 2D photographs and 3D modeling. This approach exemplifies how even partial 3D reconstruction can yield clinically relevant anthropometric indices. Alexa’s Kinect-based study [28] demonstrated that volumetric features alone can predict fat percentage with R^2^ = 0.72 and RMSE = 8.02%, even from single depth map. This finding illustrates how volumetric data, even when incomplete, can yield valuable body composition information.

#### 5.2.3. Shape Descriptors and Curvature Analysis

Advanced shape descriptors, such as surface curvature, provide anatomical insight beyond circumferential data. Laws et al. [44] demonstrated that curvature-based metrics correlate with tissue distribution. Combining localized geometric features with global statistical shape parameters improves prediction robustness [36].

#### 5.2.4. Comparison with Traditional Methods

The accuracy of voxel-based body composition assessment relative to traditional methods represents a critical consideration for clinical adoption. Research comparing 3D scan-derived measures against Dual-energy X-ray absorptiometry (DXA), hydrostatic weighing, and Bioelectrical Impedance Analysis (BIA) has shown promising results.

Alexa et al. [28] and Astorino et al. reported significant correlations between 3D-scan-derived estimates and traditional methods like BIA, DXA, and hydrostatic weighing. While promising (RMSE ~8%), voxel-based models still fall short of clinical diagnostic standards, supporting their use for monitoring rather than diagnosis.

### 5.3. Gait Integration Possibilities

#### 5.3.1. Morphology-Locomotion Relationships

The joint modeling of body shape and gait provides insight into morphology-driven movement variations. Pixel2Pose [41] illustrates this integration by generating skeletal poses from ToF depth data, enabling combined shape and motion analysis.

#### 5.3.2. Biomechanical Analysis and Clinical Applications

Volumetric models allow accurate mass segmentation, center-of-mass estimation, and joint loading computation. Tsoli et al. and Weiss et al. used parametric models animated with captured gait data for personalized biomechanical simulation, supporting clinical use in MSK disorder evaluation and rehabilitation planning [36,40].

#### 5.3.3. Longitudinal Monitoring and Intervention Assessment

By combining static 3D scans with gait analysis, clinicians can assess morphological and functional changes during interventions. Weiss et al. [40] highlight the feasibility of such monitoring using affordable sensors in clinical environments.

### 5.4. Practical Limitations and Deployment Constraints

#### 5.4.1. Segmentation Errors and Depth Artifacts

Reconstruction accuracy suffers from background blending and noise near object boundaries, especially in complex or poorly lit scenes [29]. In participants with obesity, deep tissue and surface curvature introduce shadows and occlusions that distort measurements [28].

#### 5.4.2. Resolution and Surface Quality Limitations

Although voxel super-resolution (VSR) improves detail, raw sensor limits persist [22]. Surface irregularities can reduce measurement fidelity and affect curvature-based descriptors.

#### 5.4.3. Posture Variability and Subject Positioning

Pose variability can obscure real anthropometric changes. Weiss et al. [40] mitigated this using SCAPE, allowing shape–pose decoupling. Alexa’s work [28] emphasized controlled multi-angle capture to minimize variance, though such constraints may be impractical outside lab settings.

#### 5.4.4. Clothing and Surface Appearance Effects

Loose clothing distorts contours; skin reflectance can confuse depth estimation. Research protocols typically require tight-fitting clothing. Textureless surfaces or highly reflective materials further reduce stereo depth accuracy, particularly for RealSense D435 [29].

#### 5.4.5. Accuracy Compared to Gold Standards

While depth-based reconstructions show promise (e.g., Alexa RMSE ~8% vs. BIA [28]), they are not yet equivalent to DXA or hydrostatic methods. Chuang-Yuan et al. [29] found persistent sensor-induced errors, capping achievable accuracy despite algorithmic refinement.

Voxel-based 3D body modeling using consumer-grade depth sensors offers an accessible and evolving method for anthropometric assessment. Advances in voxel super-resolution, implicit modeling, and statistical body models (SMPL, SCAPE) have enhanced accuracy and integration potential with gait analysis. However, challenges persist regarding posture variability, surface quality, and measurement accuracy compared to clinical gold standards.

Future work should focus on the following:

- Improving voxel reconstruction fidelity via better sensors and algorithms;
- Adapting models for robust real-world deployment (clothing, motion, lighting);
- Validating outcomes against reference techniques in diverse populations.

The integration of 3D modeling and gait tracking offers promising new paradigms in clinical monitoring, obesity evaluation, and physical function analysis, paving the way toward accessible, data-rich health assessments.

To provide a rigorous comparison of gait analysis, pose estimation, and voxel-based human body modeling for obesity-related research and clinical applications, Table 13 synthesizes their principal methodological distinctions. This comparative framework outlines each approach’s measurement outputs, technical and operational requirements, accuracy, and current validation status, thereby supporting informed methodological selection according to specific research aims and practical constraints.

In addition to the methodological comparison detailed in Table 14 and Table 15 presents a structured SWOT (Strengths, Weaknesses, Opportunities, and Threats) analysis of the optical capture and modeling technologies discussed in this paper. This strategic evaluation delineates the technical advantages, limitations, potential applications, and challenges associated with each modality, considering their performance characteristics, suitability across diverse deployment environments, and scalability for large-scale studies. Such an analysis is particularly pertinent for informing technology selection in longitudinal obesity research, clinical screening, and advanced biomechanical modeling, where careful consideration of trade-offs among accuracy, usability, cost, and environmental adaptability is essential for optimal implementation.

## 6. Hybrid Systems and Sensor Fusion Strategies for Obesity Detection

Recent advancements in sensor technologies, computational methods, and artificial intelligence have revolutionized approaches to obesity detection and monitoring. This part of the review examines cutting-edge research on hybrid systems and sensor fusion strategies that leverage gait analysis and human voxel modeling for more accurate, non-invasive, and accessible obesity detection. The integration of multiple sensing modalities, privacy-preserving computational approaches, explainable AI methods, and scalable deployment frameworks represents a paradigm shift in obesity management and intervention strategies.

### 6.1. Multimodal/Sensor Fusion System Architectures

Traditional approaches to obesity assessment rely primarily on anthropometric measurements such as BMI, waist circumference, and skinfold thickness. While useful, these methods provide limited insights into the functional implications of excess weight and fail to capture the complex physiological and biomechanical manifestations of obesity [31]. Gait analysis offers a complementary approach by capturing the biomechanical manifestations of obesity during walking. Early gait analysis systems typically employed single-sensor modalities, such as force plates, optical motion capture, or inertial sensors, each with inherent limitations in comprehensiveness and practical deployment.

The emergence of hybrid systems combining multiple sensor types represents a significant advancement for obesity detection through gait analysis. These multimodal approaches leverage the complementary strengths of different sensing technologies to create more robust, accurate, and practical assessment tools. By simultaneously capturing spatial, temporal, kinematic, and sometimes kinetic parameters of gait, these systems can detect the subtle and complex alterations associated with different degrees of obesity and fat distribution patterns.

Research in this domain has increasingly focused on creating affordable, accessible systems that maintain clinical-grade accuracy [24,46]. This reflects a growing recognition of gait analysis as not merely a research tool but a potential component of routine obesity screening and monitoring programs across various healthcare settings.

#### 6.1.1. Integration of Optical and Depth Sensing Technologies

The integration of RGB cameras with depth sensors has emerged as a foundational approach in obesity detection systems. Kinect-based systems have demonstrated promise by combining RGB imagery with depth information to construct accurate body morphological representations. These systems can generate detailed 3D body models that enable volumetric analysis of body segments-a critical capability for accurate obesity assessment that goes beyond simple anthropometric measurements [24]. The Microsoft Kinect sensor, in particular, has been widely adopted due to its ability to track 25 body joints in real-time while simultaneously capturing RGB and depth data streams, facilitating comprehensive gait and posture analysis in obese individuals.

Recent research demonstrates the potential of combining optical and depth sensing technologies with gait analysis for improved obesity detection and movement assessment. Depth vision sensors, when used alongside wearable sensors, enhance abnormal gait classification [47]. For obese subjects, marker-based optoelectronic systems and wearable magneto-inertial measurement units are commonly used, often integrated with force platforms [20]. An integrated system using depth sensing cameras and IMU sensors, processed through deep learning algorithms, shows significant improvements in gait data accuracy compared to single-method approaches [21]. The Intel RealSense camera, a leading 3D depth sensing technology, has demonstrated promising applications in clinical research, particularly for gait analysis and rehabilitation [30]. These combined technologies offer potential for developing more precise, objective movement-based endpoints for tracking treatment interventions in clinical trials involving obese individuals.

#### 6.1.2. Fusion of Inertial and Optical Sensors

Wearable inertial sensor systems represent another important approach for gait analysis in obesity assessment. These systems typically employ networks of IMUs containing accelerometers, gyroscopes, and sometimes magnetometers attached to various body segments. A wearable magneto-inertial system for gait analysis (H-Gait) has been specifically validated for both normal weight and overweight/obese individuals [48]. This system uses magneto-inertial sensors to capture detailed gait parameters and has demonstrated good reliability across different weight categories.

Inertial sensor systems have shown particular utility for upper limb motion analysis in individuals with obesity, revealing characteristic alterations in arm swing patterns [49]. These systems can quantify parameters such as arm swing amplitude, symmetry, and coordination with lower limb movements—metrics that have proven sensitive to weight-related changes in gait mechanics.

The advantages of wearable inertial systems include portability, ability to function in various environments, and capacity for continuous monitoring during activities of daily living. Recent miniaturization of IMU technology has led to unobtrusive sensors embedded in clothing, footwear, or accessories, enabling long-term monitoring without significant user burden. However, these systems require careful calibration, synchronization, and drift correction to maintain accuracy.

The combination of IMUs with optical sensing technologies has demonstrated superior performance in characterizing obesity-related gait patterns. IMUs provide detailed information about segment accelerations and orientations, complementing the spatial data captured by optical systems. Research has shown that fusion of these modalities allows for more accurate quantification of gait parameters [45]. At the current state, the presence of bias in the research of Cerfoglio et al. limits the applicability of the inertial-based system in clinics. further research is intended in in this context.

Lee et al. utilized smartphone cameras and wearable IMUs to estimate the knee adduction moment (KAM) and knee flexion moment (KFM), developing a model to optimally diagnose walking patterns and reduce knee load-a particularly relevant application for obese populations who experience greater joint stress during locomotion [24]. The integration of these complementary data streams provided a more holistic understanding of obesity-related biomechanical adaptations than either modality could achieve independently.

#### 6.1.3. Thermal Imaging Integration for Multimodal Assessment

Thermal imaging presents a unique opportunity to enhance obesity detection by providing information about subcutaneous fat distribution and brown adipose tissue (BAT) activity. Snekhalatha et al. demonstrated that thermal imaging of abdominal, forearm, and shank regions revealed significant temperature differences between obese and normal-weight individuals, with the abdominal region showing a 4.703% temperature difference [31]. This thermal signature can be attributed to the insulating properties of adipose tissue and altered thermogenesis in obese individuals.

When integrated with skeletal tracking and 3D body modeling, thermal data provides an additional physiological dimension to obesity assessment. Multi-stream architectures that combine thermal, RGB, depth, and inertial data have been proposed, employing various fusion strategies:

1. Early fusion: Feature-level integration that combines raw or low-level features from multiple sensors before processing;
2. Late fusion: Decision-level integration that combines independently processed data from each sensor at the decision stage;
3. Hybrid fusion: Combinations of early and late fusion approaches that leverage the strengths of each method.

Research by Lee et al. introduced a non-contact sensor system that generates 3D body models from 2D images, demonstrating how even limited image inputs (front and side views) can be synthesized into comprehensive 3D body data for obesity monitoring [24]. This approach addresses accessibility issues by reducing hardware requirements while maintaining assessment accuracy.

#### 6.1.4. Advanced Data Integration Frameworks

More sophisticated fusion architectures have emerged to handle the heterogeneous data types and sampling rates inherent in multimodal obesity detection systems. Cross-modal attention mechanisms enable systems to dynamically weight the contribution of each modality based on its relevance to specific aspects of obesity assessment. For example, thermal data might receive greater emphasis when evaluating metabolic activity, while inertial and depth data might be prioritized when analyzing gait patterns.

Recent advancements in 3D body model reconstruction have improved upon traditional point cloud techniques. SPLATNet introduces sparse bilateral convolutional layers for efficient point cloud processing, outperforming existing methods in 3D segmentation tasks [41]. Jiang et al. propose a skeleton-aware approach using PointNet++ and SMPL parameters, incorporating graph aggregation and attention modules for better feature extraction and mapping [35]. Bhatnagar et al. combine implicit function learning with parametric models, using an Implicit Part Network to predict outer and inner body surfaces from sparse point clouds [50]. Their method allows for controllable and accurate 3D reconstructions, even with clothing. Zhou et al. introduce a Gaussian Process layer and adversarial training to encode surface smoothness and shape coherence in their deep autoencoder architecture, demonstrating quantitative improvements over existing DNN-based methods for human body mesh reconstruction from point clouds [51].

### 6.2. Federated Learning and Data Privacy

Gait analysis for obesity detection inherently involves collection of sensitive biometric data that raises significant privacy concerns. Traditional machine learning approaches requiring centralized data aggregation present several problems in this context:

1. Personal Health Information Protection: Gait patterns constitute protected health information under regulations like HIPAA and GDPR, necessitating stringent data handling protocols.
2. Identification Risk: Gait is a behavioral biometric that can uniquely identify individuals, creating potential for unauthorized tracking or identification if data is compromised.
3. Stigmatization Concerns: Data relating to obesity carries social stigma risks, making privacy preservation particularly important for patient dignity and acceptance of monitoring technologies.
4. Longitudinal Data Vulnerabilities: Continuous monitoring of gait for obesity management generates extensive personal datasets that, if centralized, create attractive targets for data breaches.

These privacy challenges have historically limited widespread implementation of gait-based obesity monitoring systems, particularly in non-clinical settings like schools or community health programs. The emergence of federated learning approaches offers a promising solution to these concerns by fundamentally changing how models are trained and deployed.

#### 6.2.1. Comparative Analysis of FL Algorithms for Obesity Detection

Several federated learning algorithms have been evaluated in the context of gait-based activity recognition, with varying performance characteristics relevant to obesity detection:

1. Federated Averaging (FedAvg): The most fundamental FL algorithm works by averaging model updates received from multiple clients before updating the global model. FedAvg performs adequately in homogeneous environments where gait data distributions are similar across users. It offers the advantage of minimizing communication overhead (8.5 MB), making it suitable for resource-constrained devices. However, FedAvg struggles with convergence in heterogeneous settings where gait patterns vary significantly across users with different degrees of obesity [52,53].
2. Federated Proximal (FedProx): This extension of FedAvg addresses statistical heterogeneity in federated learning by introducing a proximal term that restricts local model updates, preventing destabilizing changes. We believe that FedProx is particularly valuable for gait-based obesity detection, where individual users may have unique walking patterns influenced by varying fat distribution, compensatory mechanisms, and comorbidities. By reducing client drift, FedProx ensures more stable learning across diverse populations [52,54].
3. SCAFFOLD (Stochastic Controlled Averaging for Federated Learning): This advanced algorithm improves upon both FedAvg and FedProx by incorporating variance reduction techniques. SCAFFOLD corrects for client drift by maintaining control variates that align local model updates with the global model’s direction. Comparative studies show SCAFFOLD achieves the highest accuracy (89.1%) and fastest convergence (70 rounds) among FL algorithms for gait analysis. It also demonstrates superior privacy preservation (0.9 privacy score) and explainability (79.4), making it particularly suitable for obesity detection systems that must balance performance with interpretability for clinical use [52].

The selection of an appropriate FL algorithm depends on specific requirements of the obesity detection system, particularly regarding trade-offs between model performance, privacy protection, and deployment constraints. Systems deployed in highly heterogeneous populations (e.g., community-wide screening programs) may benefit from SCAFFOLD’s robustness, while resource-constrained applications might prioritize FedAvg’s efficiency.

#### 6.2.2. On-Device Learning for Mobile Obesity Screening

On-device learning represents an advanced implementation of federated learning that further enhances privacy and enables real-time obesity risk assessment through gait analysis. This approach performs model training and inference entirely on the user’s device, offering several advantages for mobile obesity screening:

1. Maximum Privacy Protection: Raw gait data never leave the device, addressing concerns about collection and storage of sensitive biometric information.
2. Real-Time Assessment: Models can provide immediate feedback on obesity-related gait parameters without requiring cloud connectivity, enabling point-of-care applications.
3. Personalization with Privacy: Models can adapt to individual walking patterns while still benefiting from population-level insights through federated updates.
4. Reduced Infrastructure Requirements: By distributing computational load across user devices, on-device learning reduces need for centralized server infrastructure.

Implementation typically employs lightweight neural networks optimized for mobile processors, with techniques such as model pruning, quantization, and knowledge distillation reducing computational requirements while maintaining accuracy. Research in mobile health applications has demonstrated feasibility of deploying federated learning for health monitoring on resource-constrained devices [55].

The integration of federated learning with Internet of Medical Things architecture has shown promise for obesity risk detection. In these systems, data such as BMI and gait parameters are analyzed locally to assess obesity risk, with expert recommendations generated based on results while preserving user privacy through federated computation [55]. As mobile devices increasingly incorporate advanced sensing capabilities, the potential for widespread, privacy-preserving obesity screening through gait analysis continues to expand.

### 6.3. Scalable Deployment and Real-Time Systems

The translation of advanced obesity detection technologies from research settings to widespread clinical and community use requires careful consideration of scalability, real-time processing capabilities, and deployment strategies.

#### 6.3.1. Edge Computing Architectures for Real-Time Analysis

Real-time obesity detection requires processing complex multimodal data streams with minimal latency. Edge computing architectures that perform computation near the data source rather than in remote data centers have emerged as a preferred approach for these applications.

The custom CNN developed by Snekhalatha et al. for thermal image-based obesity classification was optimized for edge deployment, achieving real-time performance while maintaining high accuracy (92%) [31]. By distributing processing across edge devices and local servers, these systems can deliver immediate feedback during obesity screening sessions without requiring constant connectivity to cloud resources.

Optimization techniques such as model quantization, pruning, and knowledge distillation have been employed to reduce the computational requirements of obesity detection models without sacrificing accuracy. These approaches are particularly important for deployments in resource-constrained settings such as schools and community health centers.

#### 6.3.2. School-Based Implementation Strategies

Schools represent critical settings for early obesity detection and intervention. Scalable deployment in educational environments requires systems that have the following characteristics:

1. Non-invasive and respectful of privacy concerns;
2. Capable of efficiently screening large numbers of students;
3. Simple enough to be operated by school health personnel;
4. Affordable within typical school health program budgets.

Recent pilot implementations have demonstrated the feasibility of using sensor fusion approaches for school-based obesity screening. These systems typically employ a combination of depth cameras and simplified thermal imaging to assess body composition and movement patterns during physical education activities. The non-contact sensor approach developed by Lee et al. is particularly well-suited for school settings, as it requires minimal equipment and can be integrated into existing health assessment protocols [24].

Privacy considerations are especially important in school implementations, with successful deployments employing federated learning approaches that keep all identifiable data within the school’s systems while still benefiting from model improvements across multiple schools.

#### 6.3.3. Clinical Integration Frameworks

Integration of advanced obesity detection systems into clinical workflows presents distinct challenges and opportunities. Clinical deployments typically require the following:

1. Interoperability with existing electronic health record (EHR) systems;
2. Compliance with medical device regulations;
3. Integration with established clinical assessment protocols;
4. Support for longitudinal patient monitoring.

Successful clinical implementations have employed modular architectures that separate data acquisition, processing, and visualization components. This approach allows hospitals and clinics to customize deployments based on their specific needs and existing infrastructure.

The thermal imaging approach described by Snekhalatha et al. has been successfully integrated into clinical settings, with the CNN-based classification system achieving an area under the curve (AUC) value of 0.948 in distinguishing obese from normal patients [31]. This performance level makes the system suitable for clinical use as a rapid screening tool, with positive cases referred for more comprehensive assessment.

#### 6.3.4. Telemedicine and Remote Monitoring Solutions

The COVID-19 pandemic accelerated the adoption of telemedicine solutions, creating new opportunities for remote obesity monitoring and intervention. Remote monitoring systems typically leverage consumer devices such as smartphones and home cameras to collect data that would previously have required in-person clinical visits.

The approach developed by Lee et al., which generates 3D body models from simple 2D images, is particularly well-suited for telemedicine applications [24]. Patients can capture front and side images using their smartphones, with the system generating detailed body composition analyses that can be reviewed by healthcare providers during virtual consultations.

These remote monitoring solutions employ several strategies to ensure data quality and reliability:

1. Standardized capture protocols with real-time guidance;
2. Automated quality control to reject unsuitable images;
3. Calibration procedures to account for varying camera characteristics;
4. Confidence metrics that indicate measurement reliability.

The integration of these systems with telehealth platforms creates comprehensive obesity management solutions that combine detection, monitoring, and intervention components within unified user experiences.

### 6.4. Ethical Considerations in Deploying Gait and Body Modeling Technologies for Obesity Detection

The integration of gait analysis, pose estimation, and human voxel modeling technologies into obesity detection and monitoring introduces a complex array of ethical considerations that extend well beyond technical performance. As these optical sensing systems are increasingly deployed in real-world settings, such as homes, schools, and outpatient clinics, the ethical landscape expands to encompass issues of fairness, privacy, and equity, each of which must be systematically addressed to ensure responsible and equitable use.

Algorithmic Fairness: A central ethical concern is the potential for algorithmic bias arising from the underrepresentation of individuals with obesity or non-normative body types in training datasets. This can lead to misclassification, missed diagnoses, or diminished tracking performance in precisely the populations most in need of accurate assessment. From a clinical and societal perspective, such biases risk reinforcing existing health disparities and undermining trust in digital health interventions. Ensuring inclusive, morphology-aware training and validation is essential to prevent bias and maintain clinical trust.

Privacy and Consent: The collection of detailed biometric and anthropometric data in uncontrolled, everyday environments introduces substantial privacy risks. Optical sensing technologies can generate highly granular spatiotemporal movement patterns and 3D body reconstructions, which are inherently identifying and susceptible to misuse or unauthorized access. Robust privacy-preserving mechanisms—such as encrypted data pipelines, on-device processing, and federated learning—are essential, alongside clear, ongoing, and context-specific consent processes that are accessible to all users, including vulnerable populations. These measures must extend beyond initial ethics approval, ensuring meaningful user autonomy and protection throughout the data lifecycle

Equity and Accessibility: Without broad validation and equitable deployment, these technologies risk exacerbating health disparities. High-cost systems may be limited to well-resourced settings, while less accurate consumer devices may be used elsewhere without proper calibration, reinforcing inequalities. Ethical deployment therefore requires inclusive validation studies, transparency about system limitations, and implementation strategies that prioritize accessibility and do not reinforce existing inequities.

Additional Considerations: The early detection of obesity through these technologies also raises concerns about potential stigmatization, anxiety, and unintended psychological consequences, particularly in pediatric or vulnerable populations. Ethical frameworks must weigh the benefits of early intervention against the risks of harm, ensuring that positive outcomes demonstrably outweigh potential negative effects.

In summary, the responsible deployment of gait and body modeling technologies for obesity detection hinges on proactive strategies to mitigate algorithmic bias, safeguard privacy, and promote equity in access and performance. Table 16 provides a comparative overview of how the reviewed studies address (or fail to address) these ethical dimensions, underscoring the need for standardized benchmarks and ongoing ethical oversight in the development and implementation of obesity-related sensing systems

In conclusion, the integration of hybrid systems and sensor fusion strategies for obesity detection represents a significant advancement over traditional assessment methods. By combining multiple sensing modalities-including optical, depth, inertial, and thermal technologies-these systems provide more comprehensive and accurate characterizations of obesity-related physiological and biomechanical alterations. The incorporation of federated learning approaches addresses critical privacy concerns while enabling continuous model improvement, while explainable AI techniques translate complex sensor data into clinically actionable insights. Scalable deployment architectures facilitate the implementation of these technologies across diverse settings, from schools to clinics to home environments, creating new opportunities for early intervention and ongoing management of obesity.

Future research directions should focus on further integration of metabolic and behavioral sensing modalities, refinement of privacy-preserving learning techniques, development of more intuitive explanatory frameworks, and validation of these systems in diverse real-world settings. As these technologies mature, they have the potential to transform obesity detection and management from periodic clinical assessments to continuous, personalized monitoring and intervention.

## 7. Future Directions and Research Opportunities for Obesity Detection Based on Gait Analysis

In today’s rapidly evolving landscape, optical sensor-based methods for obesity assessment are opening new doors for non-invasive, portable, and scalable solutions that can be deployed in both clinical and everyday environments. To unlock their full potential for clinical translation, it is crucial to not only track technological advances, such as improvements in sensor accuracy, edge-AI processing, and robust pose estimation algorithms, but also to consider the practical realities of deployment. This includes addressing factors like user accessibility, integration with existing healthcare workflows, and data privacy concerns. Moreover, navigating the regulatory landscape is essential to ensure these innovations meet safety and efficacy standards for widespread adoption. By fostering interdisciplinary collaboration and focusing on real-world validation, future research can bridge the gap between laboratory innovation and impactful, patient-centered care.

### 7.1. Toward Portable, AI-Enabled Obesity Detection

Making optical sensing systems more portable and embedding artificial intelligence directly on devices are key steps toward expanding obesity screening beyond the walls of traditional clinics. Recent breakthroughs have shown that single-smartphone video pose-estimation frameworks, such as MediaPipe [37] and OpenPose [14], can accurately recognize body poses in real time using only the modest processing power of a mobile device. Similarly, compact RGB-D scanners like Intel’s RealSense D435, Kinect and other affordable sensors are now capable of reconstructing body shape and volume on the fly, offering an effective compromise between measurement accuracy and portability [29].

To provide a structured overview of the methodological landscape in obesity detection using optical sensing technologies, the following Table 17 summarizes key characteristics from recent studies. It focuses on two main categories: (i) single-smartphone video pose estimation and inertial sensor-based systems, and (ii) compact RGB-D scanners. For each, the table details reported sample sizes, accuracy metrics (such as RMSE, classification accuracy, and error margins), and specific deployment contexts. This synthesis helps clarify the current state of research and supports comparative analysis across system types and use cases in both clinical and real-world environments.

Looking ahead, future research should prioritize optimizing optical sensor-based systems for practical clinical use by validating their performance across diverse patient populations and ensuring seamless integration with telehealth platforms, an increasingly important consideration in contemporary healthcare.

The successful adoption of these technologies requires not only ongoing technological innovation but also strict adherence to regulatory standards, robust data governance, and interoperability with existing health IT infrastructure. Compliance with medical device regulations (such as FDA SaMD and CE marking), privacy laws (including GDPR and HIPAA), and interoperability standards (e.g., HL7 FHIR, DICOM) is essential to guarantee safety, effectiveness, and secure, seamless data exchange. Proactively addressing these regulatory, privacy, and interoperability requirements will accelerate the safe and effective implementation of optical sensor-based systems in clinical practice.

The integration of these technologies offers a significant opportunity to expand the accessibility and scalability of obesity screening. By enhancing portability and embedding real-time, on-device intelligence, such systems could enable low-cost, accurate, and non-intrusive monitoring outside traditional clinical environments. This advancement paves the way for broader clinical translation, especially in remote or underserved communities, and supports inclusive, continuous, and ecologically valid health monitoring.

Nevertheless, several critical challenges remain to be addressed for successful clinical translation. Battery drain and high computational demands currently limit the feasibility of prolonged mobile use. Motion and soft tissue artefacts, particularly prevalent in obese individuals, can compromise measurement accuracy, while footwear compliance affects the consistency of gait analysis. Additionally, regulatory hurdles and the lack of standardized validation procedures continue to impede clinical adoption. Overcoming these obstacles is essential to ensure robust, ethical, and generalizable implementation in real-world healthcare settings.

In summary, by advancing portability and on-device intelligence, optical sensor-based technologies have the potential to make obesity screening more accessible, scalable, and responsive to real-world clinical needs [28,29].

### 7.2. Standardized Protocols and Open Datasets

Current research suffers from fragmented methodologies, as evidenced by a 2024 meta-analysis identifying significant heterogeneity in gait parameter reporting across 14 obesity studies [57]. Establishing annotated obesity gait libraries with ground-truth validation requires multidisciplinary collaboration to define the following:

1. Unified spatiotemporal parameter definitions;
2. Standardized BMI classification thresholds;
3. Age- and sex-specific normative ranges.

The Health&Gait dataset represents a pioneering effort in this direction, comprising 1564 video samples from 398 participants with synchronized anthropometric and gait data [25]. However, critical gaps persist in pediatric populations, where obesity-induced gait modifications differ substantially from adults. A 2025 intervention study in obese children highlighted the need for youth-specific benchmarks, demonstrating unique pelvic kinematic adaptations during walking [11].

Open challenges include reconciling optical motion capture with wearable sensor outputs and developing cross-modal calibration protocols. Shared benchmarks must account for ethnic diversity, socioeconomic factors, and comorbid conditions to avoid algorithmic bias in heterogeneous populations.

To conclude, standardizing protocols and expanding open, well-annotated datasets represent pivotal opportunities for advancing obesity-related gait analysis toward clinical translation. Key priorities include the establishment of unified definitions for gait parameters, harmonized BMI thresholds, and age- and sex-specific normative values, with particular attention to pediatric cohorts. Additionally, integrating optical and wearable sensor systems through rigorous cross-modal calibration, alongside ensuring population diversity within benchmark datasets, will be critical for developing robust, generalizable, and clinically meaningful assessment tools.

### 7.3. Wearable and Optical Sensor Integration

Multimodal sensor fusion approaches are overcoming the limitations of single-modality systems. The INDIP platform exemplifies this trend, combining plantar pressure insoles, inertial measurement units (IMUs), and time-of-flight distance sensors to achieve ≤0.06 m stride length error across diverse cohorts including Parkinson’s and COPD patients [56]. Integrating camera-derived kinematic data with wearable heart rate (HR) and IMU metrics enables holistic health monitoring—a concept validated in video-based systems achieving 94% classification accuracy using gait features [25].

Emerging technologies leverage computer vision to extract 3D joint kinematics from smartphone videos, bypassing the need for marker-based systems. When combined with wearable-derived cardiovascular metrics, these systems can correlate gait abnormalities with metabolic parameters like VO2 max. However, lighting variability and occlusion remain technical hurdles, necessitating advanced neural networks trained on augmented datasets simulating real-world conditions.

In summary, Multimodal sensor fusion presents significant potential for comprehensive movement and health assessment by integrating camera-based kinematic data with wearable cardiovascular and inertial measurements, thereby enhancing accuracy and enabling holistic monitoring. To realize scalable and clinically viable solutions, future research should prioritize addressing challenges such as visual occlusion and lighting variability. This can be achieved through the development of advanced neural networks trained on diverse and augmented datasets.

### 7.4. Personalization with Digital Twins

Patient-specific digital twin models are revolutionizing intervention planning by simulating gait adaptations underweight change scenarios. A 2025 kinetic study demonstrated the predictive value of such models, showing improved pelvic kinematics in obese children following six-month exercise programs [11]. These virtual replicas integrate the following:

- Biomechanical body composition profiles;
- Muscle activation patterns;
- Joint loading characteristics.

Deep learning architectures trained on longitudinal gait data can forecast individualized responses to dietary, surgical, or exercise interventions. For instance, transformer-based models show promise in predicting post-bariatric surgery gait normalization trajectories using preoperative spatiotemporal parameters [57]. Federated learning frameworks enable model refinement across institutions while maintaining data privacy—a crucial consideration for sensitive health data.

The convergence of wearable technologies, advanced analytics, and personalized modeling heralds a new era in obesity detection and management. Realizing this potential requires sustained investment in standardized datasets, interoperable sensor platforms, and validation studies across diverse populations. Priorities include expanding pediatric gait databases, developing ethical AI governance frameworks, and translating laboratory innovations into scalable public health solutions. By addressing these challenges, gait analysis may soon become a cornerstone of precision medicine approaches to obesity [6,11,25,27,56,57,58].

Finally, digital twin technologies hold strong potential for personalized obesity intervention by simulating individual gait responses to treatment. Integrating biomechanical, neuromuscular, and spatiotemporal data enables predictive modeling of intervention outcomes. Future directions should prioritize federated learning frameworks, ethical AI implementation, and the expansion of pediatric datasets to support scalable, privacy-preserving, and clinically actionable digital twin applications.

## 8. Conclusions

The rapid evolution of optical sensor technologies is fundamentally reshaping the landscape of gait analysis and obesity assessment. This review has examined the methodological advances, clinical applicability, and translational challenges of state-of-the-art optical sensor systems, ranging from markerless video analytics and RGB-D cameras to hybrid multi-sensor approaches and AI-driven analytics.

Our synthesis demonstrates that modern optical systems spanning low-cost depth cameras, smartphone-based solutions, and sophisticated hybrid arrays are increasingly capable of capturing clinically relevant biomechanical and anthropometric markers. Markerless pose estimation frameworks (e.g., OpenPose, MediaPipe) and infrared depth sensors now enable real-time, non-invasive extraction of kinematic features, supporting early detection and longitudinal monitoring of obesity-related gait deviations in diverse, real-world environments. Hybrid sensor configurations further enrich biomechanical profiling and may enhance diagnostic precision, particularly for complex or subtle gait alterations.

Despite these promising developments, several barriers remain. The generalizability and robustness of current algorithms are constrained by limited representation of diverse and obese populations in training datasets, environmental variability, and lack of standardized validation protocols. Ethical considerations, especially around data privacy, consent, and algorithmic fairness, are increasingly critical as these technologies move toward widespread deployment.

**Figure 4:**
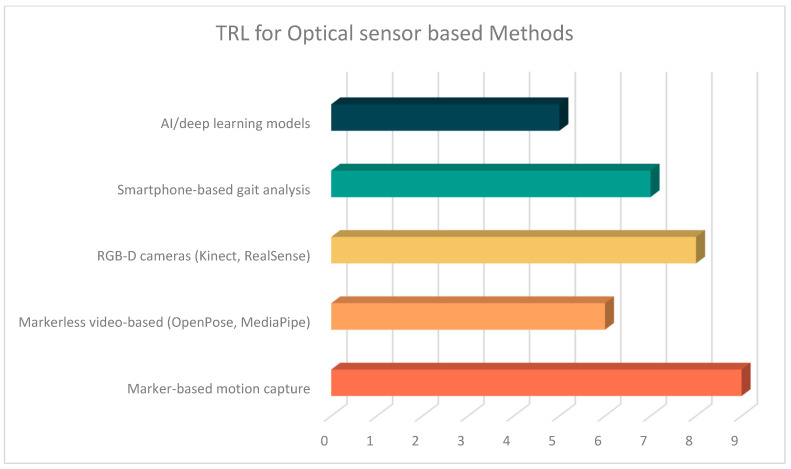
Technology readiness level of reviewed optical sensor-based methods (1 = basic principles, 9 = full deployment).

To contextualize the translational status of these technologies, Figure 4 summarizes the Technology Readiness Level (TRL), and Table 18 describes the real-world deployment status of each principal method reviewed.

**Technology Readiness Level (TRL)**

This comparative overview underscores that while traditional marker-based systems remain the clinical gold standard, emerging markerless, RGB-D, and hybrid solutions are progressing rapidly toward broader adoption. However, their full clinical and societal impact will depend on sustained efforts in validation, standardization, ethical deployment, and user-centered design.

**Future research priorities should include the following:**

- Expanding validation across diverse and pediatric populations.
- Developing and adopting standardized benchmarking protocols.
- Ensuring transparency, explainability, and fairness in AI-driven analytics.
- Integrating optical sensors with wearable and mobile health technologies for holistic, continuous monitoring.
- Addressing ethical, privacy, and data governance challenges through robust frameworks.

By aligning technical innovation with clinical needs and ethical imperatives, optical sensor-based gait analysis systems are poised to transform obesity diagnostics, enabling dynamic, individualized, and actionable health insights at scale. This evolution not only enhances early detection and personalized intervention but also paves the way for accessible, community-based public health solutions in the global fight against obesity.

## Figures and Tables

**Table 1:** The Primary databases used for the literature review study.

Database	Rationale for Inclusion	Field Coverage
PubMed/MEDLINE	Core biomedical literature	Medicine, biomechanics, clinical validation
Scopus	Broad multidisciplinary coverage	Engineering, computer science, healthcare
IEEE Xplore	Engineering and computing focus	Signal processing, sensor design, algorithms
ACM Digital Library	Computing research	Computer vision, machine learning
ScienceDirect	Multidisciplinary science platform	Optical engineering, biomechanics
Web of Science	Citation tracking capability	Cross-disciplinary research
Google Scholar	Grey literature and technical reports	Emerging technologies, pre-prints

**Table 2:** Detailed search strategy for PubMed.

Concept	Search Terms
**Population**	(“obesity” [MeSH Terms] OR obes * [TIAB] OR overweight [TIAB] OR “body composition” [MeSH Terms] OR “body volume” [TIAB] OR “body fat” [TIAB])
**Intervention-Technology**	(“OptoGait” [TIAB] OR “OpenPose” [TIAB] OR “MediaPipe” [TIAB] OR “DeepLabCut” [TIAB] OR “Azure Kinect” [TIAB] OR “Kinect v2” [TIAB] OR “RGB-D camera” [TIAB] OR “voxel modeling” [TIAB] OR “3D reconstruction” [TIAB] OR “Depth Camera” [MeSH Terms] OR “Motion Capture” [TIAB] OR “markerless motion capture” [TIAB] OR “pose estimation” [TIAB] OR “3D scanning” [TIAB] OR “voxel modeling” [TIAB])
**Outcomes**	(“Gait” [MeSH Terms] OR gait [TIAB] OR “Gait Analysis” [TIAB] OR “spatiotemporal gait” [TIAB] OR “stride length” [TIAB] OR “joint angles” [TIAB] OR “body pose” [TIAB] OR posture [MeSH Terms] OR “body reconstruction” [TIAB] OR anthropometr * [TIAB] OR “body scan” [TIAB])
**Study Design**	(“Validation Studies as Topic” [MeSH Terms] OR validation [TIAB] OR performance [TIAB] OR accuracy [TIAB] OR “technical evaluation” [TIAB] OR experimental [TIAB] OR observational [TIAB])

**Table 3:** The inclusion and exclusion criteria.

PICOS Element	Inclusion Criteria	Exclusion Criteria	Rationale
**Population**	- Human participants of any age, sex, or ethnicity - Individuals classified as having obesity (BMI ≥ 30 kg/m^2^) or overweight (BMI 25–29.9 kg/m^2^) - Studies including normal-weight participants	- Animal studies - Studies using exclusively synthetic datasets or simulations without human validation	To ensure clinical relevance and applicability to human obesity detection while allowing for comparative analyses
**Intervention/Technology**	- Optical sensing technologies (e.g., RGB cameras, depth sensors, infrared sensors) - Vision-based pose estimation frameworks (e.g., OpenPose, MediaPipe, DeepLabCut) - 3D body reconstruction/voxel modeling systems (e.g., Kinect, RealSense) - Multi-camera arrays for motion capture - Light-based gait analysis systems (e.g., OptoGait)	- Studies using exclusively contact-based or wearable sensors (e.g., accelerometers, pressure insoles) - Manual visual observation without technological augmentation - MRI, CT, or DXA-only approaches without optical sensing component	To focus specifically on non-contact optical sensing methodologies while excluding technologies that do not utilize optical principles
**Comparison**	- Studies comparing optical methods to gold standard measures (e.g., force plates, marker-based motion capture) - Studies comparing multiple optical sensing approaches - Studies comparing optical methods to traditional anthropometric measurements - Studies without explicit comparisons but with robust validation	- Studies without any validation against established methods - Studies without performance metrics or statistical validation	To ensure methodological rigor and establish validity of the optical approaches being evaluated
**Outcomes**	- Gait parameters (e.g., stride length, cadence, toe clearance) - Body shape/volume metrics - Anthropometric measurements - Joint kinematics - Biomechanical parameters - System usability and feasibility metrics	- Studies reporting exclusively on system development without outcome measures - Studies without quantifiable results	To focus on clinically relevant parameters that relate to obesity detection and assessment
**Study Design**	- Peer-reviewed journal articles - Full technical papers with empirical data - Validation studies - Observational studies - Experimental studies - Cross-sectional and longitudinal designs - Studies published between January 2000 and March 2025 - English language publications	- Conference abstracts without full papers - Editorials, commentaries, or opinion pieces - Literature reviews (used only for backward citation tracking) - Studies with serious methodological flaws - Studies without clear methodology description	To ensure inclusion of high-quality, empirical research with sufficient methodological detail

**Table 4:** Quality assessment criteria used for evaluation in this work.

Domain	Assessment Criteria	Scoring
Study Design	- Clear research objectives and questions - Appropriate study design for objectives - Adequate sample size with power analysis where appropriate	0–3 points
Participant Selection	- Clear inclusion/exclusion criteria - Representative sample of target population - Appropriate participant characteristics reported (age, sex, BMI, health status)	0–3 points
Technical Methodology	- Detailed description of hardware specifications - Comprehensive explanation of algorithms and processing pipelines - Appropriate calibration and validation procedures - Clearly defined parameters and metrics	0–4 points
Reference Standard	- Use of appropriate gold standard or reference measures - Proper implementation of reference measures - Blinding between test and reference standard where applicable	0–3 points
Data Analysis	- Appropriate statistical methods - Proper handling of missing data - Appropriate performance metrics reported (e.g., accuracy, precision, recall) - Consideration of confounding variables	0–4 points
Results Reporting	- Complete reporting of all planned outcomes - Appropriate presentation of results (tables, figures) - Comprehensive discussion of limitations - Disclosure of conflicts of interest	0–4 points
Applicability	- Relevance to obesity detection - Discussion of clinical or practical implications - Assessment of implementation feasibility	0–3 points

**Table 5:** Included studies by study type.

Study Type	Count
Validation	15
Experimental	7
Modeling	7
Pilot	6
Technical	4
Review	3
System Design	3
Anthropometric	3
Comparative	2
Systematic Review	2
Technical Validation	1
Observational	1
Cross-sectional	1
Dataset	1

**Table 6:** Technical and clinical validation summary (sample).

Title	Year	Technology Used	Validation Method	Outcome Measures	Obesity-Specific?
Exploring the Influence of BMI on Gait Metrics [5]	2025	Optical (Depth Camera)	Gait comparison across BMI	Stride, step time, stability	Yes
Deep Learning-Based Obesity Identification System [6]	2024	Smartphone Inertial Sensors	ML-based classification	Obesity detection	Yes
Gait Analysis Methods Overview [7]	2014	Wearable + non-wearable	Narrative methodology comparison	General gait metrics	No
OptoGait Motion Analysis in Young Adults [8]	2020	OptoGait	Intra-rater repeatability	Spatiotemporal gait parameters	No
Privacy-Preserving Abnormal Gait Detection [9]	2021	Computer Vision	Proof-of-concept validation	Anomaly detection	No
Functional Gait and Obesity Correlation [10]	2021	Motion capture system	Obesity vs. control group	Spatiotemporal parameters	Yes
Comprehensive Gait Analysis in Obese Children [11]	2025	Marker-based + camera	Cross-sectional study	Kinetics, gait phases	Yes
DeepLabCut Markerless Pose for Gait [12]	2025	DeepLabCut	Pose estimation accuracy	2D joint trajectories	Yes
Pose Tracking with Azure Kinect [13]	2020	Azure Kinect	Comparison with gold standard	Joint angles, stride length	Yes
3D Markerless Motion with OpenPose [14]	2020	OpenPose	Accuracy benchmark	Joint position error	No

**Table 7:** Gait features associated with obesity.

Gait Feature	Obesity-Related Alteration
**Step Width**	Increased; wider base enhances mediolateral stability
**Step Length**	Often increased in adults; variable in children; compensatory for stride control
**Walking Velocity**	Decreased; reduced speed reflects cautious gait pattern
**Stance Phase Duration**	Prolonged; greater time spent in stable double-limb support
**Double-Limb Support Time**	Increased; enhances static balance
**Single-Limb Support Time**	Decreased; minimizes demand on each limb
**Swing Phase Duration**	Reduced; contributes to shorter single-limb support
**Hip Flexion**	Excessive across gait cycle; compensatory for lower limb inertia
**Hip Movement (Frontal Plane)**	Increased lateral sway; linked to trunk mass and pelvic instability
**Knee Position (Stance)**	Slightly more extended; reduces joint torque and energy demand
**Ankle Position (Initial Contact)**	More plantarflexed; altered loading strategy
**Ankle Range of Motion**	Increased in adults; decreased dorsiflexion in obese children
**Midfoot Loading**	Elevated plantar pressure and contact area; especially midfoot
**Plantar Pressure**	Increased peak pressure and force-time integral
**Forefoot Contact Phase**	Prolonged, particularly in right foot; altered rollover mechanics
**Heel-Off and Step Duration**	Increased; contributes to overall gait cycle elongation
**Hip/Knee/Ankle Moments**	Higher joint moments; increased mechanical demand across lower limbs
**Gait Stability**	Decreased; reflects dynamic instability and fall risk

**Table 8:** Comparative overview of optical sensor technologies for gait and obesity analysis (based on provided sources).

System Type/Feature	Principles and Hardware Configuration	Accuracy	Sensitivity	Applications for Obesity Research	Technical Advantages	Technical Limitations
**Marker-Based Optical Motion Capture (OMC) Systems**	- Utilize multiple cameras (stereophotogrammetry) to track reflective markers placed on anatomical landmarks [15,19]. - Compute joint positions and segment orientations through 3D marker localization [15]. - Often integrated with force platforms to assess GRF and moments [6]. - Examples: Vicon, Qualisys (typically at 200 Hz) [19,20]. - May use full-body marker sets (e.g., 44 markers, four clusters for 6 DoF models) [19].	- Regarded as the gold standard in motion capture [12,21]. - High accuracy and repeatability [19]. - Errors up to ~9° for knee and ankle kinematics when compared with biplanar videoradiography [19].	- Not always explicitly quantified; however, high sensitivity is implied due to system precision and benchmark status as gold standards [15].	- Quantitative gait analysis to detect biomechanical alterations due to overweight/obesity. - Assessment of joint kinematics/kinetics, angular momentum, and trunk-pelvis coordination. - Evaluation of soft tissue work, metabolic cost, and risk factors in obese populations.	- High precision in kinematic and kinetic measurement [6,7,19]. - Comprehensive data capture in controlled settings [7]. - Capable of multidimensional feature extraction. - High repeatability and minimal external interference.	- High cost and laboratory confinement [6,20]. - Time-intensive setup and marker placement [15]. - Requires technical expertise [15]. - Affected by soft tissue artifacts, marker placement variability, and joint axis crosstalk [20]. - Limited platform size and constrained walking space [7].
**Markerless Motion Capture Systems (General)**	- Employ computer vision to estimate body pose and joint orientation from images or video [22]. - Leverage deep learning for 2D/3D reconstruction [23,24,25]. - Systems may use single RGB/RGB-D or multi-view camera setups [19]. - Techniques include silhouette analysis, semantic segmentation, and optical flow [9,19]. - Examples: DeepLabCut, MediaPipe Pose [26].	- Variable accuracy across systems and conditions [19]. - DeepLabCut shows good to excellent agreement in adults, less so in toddlers. - Trunk kinematics show RMSEs of 5–10°, poorer for neck [19,23]. - Some models overestimate stride/step length due to pose estimation limitations [19].	- Inferred from studies reporting high classification accuracies (e.g., CNN-LSTM models up to 97%) in obesity detection [6].	- Applied in gait analysis and obesity classification frameworks [6,25]. - Used for body model reconstruction from images, BMI estimation, and gait-based diagnosis [24,27]. - Integrated in mobile monitoring, telehealth, and computer-aided obesity diagnostics [24,27].	- Non-invasive and cost-effective [20,24,25]. - Eliminates the need for physical markers [24]. - Scalable and suitable for real-world use. - Enables automated, privacy-preserving assessment [9].	- Performance varies with lighting, occlusion, and viewpoint. - Data quality can suffer from pose estimation errors and “spikes” [19]. - High computational demands mainly for stereoscopic calculations and complex algorithms. - Volumetric and resolution limitations affect full-body accuracy [28].
**Markerless Motion Capture Systems: Depth Cameras (e.g., Microsoft Kinect, Intel RealSense)**	- Operate using Time-of-Flight (ToF) or stereoscopic methods to generate depth maps and 3D pose estimates [13,29]. - Examples: Kinect V2 (ToF) and RealSense D435 (stereoscopic) [29]. - Kinect V2 combines RGB (1920 × 1080) and IR (512 × 424) imaging at 30 Hz [13]. - Tracks up to 6 users with 25 joints each [13].	- ToF sensors provide more accurate 3D reconstruction than stereoscopic ones [29]. - Azure Kinect shows improved spatial gait parameter accuracy compared to Kinect V2 [13]. - Mean height error from Kinect point clouds: −1.12 cm [28].	- Not explicitly defined, but higher spatial accuracy implies improved sensitivity for gait-related applications.	- Applied in body fat estimation using 3D depth maps [28]. - Used in gait monitoring during home-based rehabilitation [13,19]. - Supports development of digital endpoints in clinical trials.	- Lower cost compared to OMC systems [19]. - Markerless and user-friendly. - Suitable for home and tele-rehabilitation contexts. - Enables skeletal tracking and spatial gait assessment [28,30].	- Accuracy in live human measurement remains under-explored. - Sensitive to lighting, occlusions, and viewpoints [19]. - Performance may vary due to computational load and sensor limitations.
**Photoelectric Cell Systems (e.g., OptoGait)**	- Use parallel bars with infrared LEDs to detect foot contacts. - Measure spatial and temporal gait parameters by detecting interruptions in infrared signals. - Example: OptoGait (1000 Hz, 1.041 cm resolution) [19] and MuscleLAB photocells (2 ms resolution) [25].	- High accuracy and repeatability for spatiotemporal gait variables [19,25]. - Comparable cadence and speed to camera-based systems, with some overestimation of stride/step lengths [19].	- Not explicitly reported; sensitivity inferred from clinical reliability.	- Used for assessing gait parameters and participant speed. - Frequently used in standard plantar pressure and gait analysis protocols [15].	- High temporal/spatial resolution. - Simplified setup. - Suitable for clinical assessment under professional supervision.	- Limited spatial analysis capabilities. - Higher cost than basic setups. - Restricted step capture length, requiring extended trials for reliable statistics.

**Table 9:** Architectural variants of convolutional models in gait and obesity analysis.

Architecture	Input Type	Primary Application
1D Convolutional Neural Networks (1D CNNs)	Time-series data (e.g., inertial signals)	Classification and analysis of gait patterns from sensor signals (e.g., accelerometers)
2D Convolutional Neural Networks (2D CNNs)	Image data	Human pose estimation, body part segmentation, thermal image classification
3D Convolutional Neural Networks (3D CNNs)	Volumetric data (e.g., voxels, point clouds)	3D human body reconstruction, voxel super-resolution, volumetric feature learning
Graph Convolutional Networks (GCNs)	Graph-structured data (e.g., skeletal graphs, point clouds)	Joint dependency modeling, shape estimation, and advanced pose recognition

**Table 10:** Traditional machine learning models used in gait and obesity analysis.

Model	Description	Application Context
Decision Tree [9,27]	Rule-based classifier that recursively partitions data using feature thresholds.	Classification of gait characteristics and weight-related categories.
Multilayer Perceptron (MLP) [24,25]	Feedforward neural network with one or more hidden layers.	Sensor-based gait classification and physiological prediction tasks.
Support Vector Machine (SVM) [9,16,27]	Separates data classes using optimal hyperplanes in feature space.	Used for BMI estimation and obesity classification from gait features.
Random Forest [5,9,27]	Ensemble learning technique that aggregates multiple decision trees for improved accuracy.	Recognition of movement patterns and spatiotemporal gait features.
k-Nearest Neighbor (k-NN) [9,27]	Instance-based learner that classifies based on feature proximity to labeled examples.	Pattern matching in gait signals from wearable sensors.
Logistic Regression [27]	Statistical model used for binary or multi-class classification tasks.	Body type and gait pattern classification using extracted features.
Bayesian Regularization Artificial Neural Network (BRANN) [21]	Neural network enhanced with regularization to prevent overfitting.	Sensor fusion and classification in multi-modal obesity detection tasks.

**Table 11:** Comparative summary of pose estimation algorithms: MediaPipe, OpenPose, and DeepLabCut.

Metric/Feature	MediaPipe [26]	OpenPose [14,19,32,34]	DeepLabCut [12,19,33,39]
**Keypoints**	33 real-time 3D keypoints (incl. face, hands, feet) [37]	25 2D keypoints (Body25 model)	User-defined body parts
**Speed/Efficiency**	Inference time: 0.774 ms on Samsung S23 UltraModel size: 3.14 MB + 12.9 MB	Real-time multi-person 2D pose estimation using a 10-layer VGG19 network	Requires ~200 labeled images to train
**Accuracy**	Described as high; no specific RMSE/PCK reported	MAE: 47% < 20 mm 80% < 30 mm 10% > 40 mm	RMSE: Human labeler: 2.69 ± 0.1 px Joint angles: - Hip: 0.1–10.5° - Knee: 0.7–3.9° - Ankle: 0.7–3.9° Trunk: 5–10° RMSE Neck: poor validity
**Limitations**	Input optimized for 256 × 256 pxPerformance varies on non-Snapdragon devices	2D tracking errors: object misidentification, segment confusion	Suboptimal for distal foot keypointsError propagation in 3D estimation
**PCK**	Not reported	Not directly provided	Median test error: 2.69–5.62 px DeeperCut (which DeepLabCut is derived from) achieved 58.7% Average Precision (MPII Multi-Person dataset)
**mAP (mean** Average Precision)**/mPCP** (mean Percentage of Correct Parts)/**AOP** (Average Over Parts)	Not reported	Not reported	showed significant improvements in mPCP (mean Percentage of Correct Parts) and AOP (Average Over Parts) on the WAF dataset compared to DeepCut.
**MPTPE**	Not reported	Not reported	Not directly reported, but RMSE and joint errors used as equivalents

**Table 12:** Summary of Accuracy and Limitations of OpenPose-Based Markerless Motion Capture.

Aspect	Findings
**Accuracy (MAE < 20 mm)**	~47% of all calculated mean absolute errors
**Accuracy (MAE < 30 mm)**	~80% of errors fall below this threshold
**High Error Rate (MAE > 40 mm)**	~10% of errors
**Primary Cause of High Errors**	Failures in OpenPose’s 2D tracking
**Examples of Tracking Failures**	- Misidentifying objects as body segments - Confusing one body segment with another
**Implication for Markerless Systems**	Reasonable accuracy for many applications, but limited robustness in diverse conditions
**Comparison to Marker-Based Systems**	Can approach similar accuracy, but with notable tracking limitations

**Table 13:** Comparison of voxel-based and 3D shape reconstruction methodologies.

Methodology	Principles	Key Algorithms/Mechanisms	Volumetric Error/ WHR Estimation Accuracy
**Traditional Voxel-Based Methods**	Converts irregular point clouds into 3D volumetric grids; early methods used fixed voxel grids; recent advances use hierarchical and sparse voxel grids [41].	3D CNNs over voxel grids; adaptive subdivision for higher surface resolution [41].	High memory/computation cost limits resolution. No specific error or WHR values; resolution insufficient for fine anatomical detail [41].
**SPLATNet (SParse LATtice Networks)**	Processes point clouds directly without voxelization by projecting onto a sparse lattice [41].	Bilateral Convolution Layers (BCLs) on a permutohedral lattice; integrates 3D (SPLATNet3D) and 2D (SPLATNet2D-3D) features [41].	Avoids voxel discretization artifacts [41]. preserves detail. While specific numerical volumetric errors or WHR accuracies are not provided, its design principles suggest an improvement in preserving surface detail compared to traditional voxelization [24,41].
**Voxel Super-Resolution (VSR) + MF-PIFu**	VSR is part of a coarse-to-fine methodology for reconstructing detailed 3D human body models from multi-view images. A coarse 3D model is initially estimated (using MF-PIFu) then voxelized into a low-resolution voxel grid. VSR then refines this low-resolution grid by learning an implicit function [22].	Coarse Stage (MF-PIFu): Learns a Pixel-aligned Implicit Function based on Multi-scale Features (MF-PIFu) VSR Refinement Stage: Takes the low-resolution voxel grids as input and refines them using a multi-stage 3D convolutional neural network to extract multi-scale features [22].	VSR is quantitatively evaluated using metrics such as Point-to-surface error (P2S), Chamfer-L2, and Intersection over Union (IoU): - CAPE Dataset: P2S ↓ from 0.9428 to 0.4954 cm, Chamfer-L2 ↓ from 0.0196 to 0.0062 cm, IoU ↑ to 84.40% [22]. - Articulated Dataset: P2S ↓ to 0.3754 cm, Chamfer-L2 ↓ to 0.0032 cm, IoU ↑ to 90.51%. WHR accuracy not directly addressed [22].
**KinectFusion (Voxel-Configurable)**	Generates 3D point clouds from consumer depth cameras by converting captured depth data into a 3D volumetric representation where a voxel resolution can be set [29].	KinectFusion techniques with inputs from depth cameras. The process involves random sample consensus algorithms and density filters for selecting regions of interest, and the iterative closest point (ICP) algorithm for aligning generated point clouds to a reference model [29].	The resolution of KinectFusion was set to 256 voxels/m, with tests also conducted at 128, 384, and 512 voxels/m to evaluate its effect. Best case 3D error: 2.0 mm (ToF vs. stereo). This error can lead to ~2.0 cm girth deviation. No direct WHR data, but the potential for 2.0 cm variation in girth measurement suggests an influence on body circumference estimations [29].
**PointGAN and 3D-R2N2** [24]	Generate 3D bodies from 2D inputs using deep generative models.	Point-based GAN and recursive CNNs over voxel grids.	Severe limitations beyond 64^3^ voxels; 10× training time increase; cannot derive circumferences or WHR, excluded from some studies => This implies that they were practically unusable or highly inaccurate for detailed body measurements like WHR [24].
**LS3D (Photonic Scanning)** [42]	Uses 3D photonic scanner to generate mesh. Circumferential measurements are then computed by defining 2D planes that intersect this 3D model. While not a voxel method, it deals with 3D shape reconstruction for body measurements.	Polygonal mesh from scan → calculate linear distances → circumference calculated by summing distances along slices. Proprietary algorithms then use these measurements to output body circumferences, WHR, and body fat percentage (BF%).	WHR: r = 0.81 with Gulick tape, LoA = ±0.06, 87.1% within RCI = 0.04. Poor for absolute waist/hip values, good for ratio. Volumetric error not specified.
**Model-Based Anthropometry** [36]	Fits deformable 3D body model to scan, predicts (using regularized linear regression) measurements using shape features. While not strictly voxel-based, it directly relates to 3D shape reconstruction and measurement.	Registers scan to parametric shape model → computes local/global features → regularized regression.	The method’s accuracy is evaluated using the Mean Absolute Difference (MAD) and Average Mean Absolute Difference (AMAD): AMAD ≈ 1 cm (1.2–1.3 × ANSUR error). 10–15% lower error than commercial tools. WHR not isolated but circumferences included in overall error metrics.

**Table 14:** Comparative summary of motion-capture methodologies relevant to obesity research.

Attribute	Gait Analysis	Pose Estimation	Human Voxel Modeling
**Typical setup**	Marker-based Optical motion Capture (OMC) and IMUs.	Markerless (RGB, RGB-D, Depth Cameras).	3D Reconstruction from Depth Cameras/Images
**Measurable Markers**	Reflective markers (e.g., 39–65) [13,19] or IMUs placed on body segments [17,45]. Force plates, pressure insoles [6].	Keypoints (e.g., joints) extracted via deep learning; 2D/3D skeletons [19].	3D point clouds [29], voxel models, anthropometric landmarks [36].
**Accuracy (Typical Error)**	OMC: High (0.15–2 mm position error) [13]. IMU: Good agreement kinematic measures (ICC 0.80–0.97) but spatiotemporal it is less consistent [17].	Mean differences for lower limb joint angles in [19]: 0.1–10.5° for hip (3 DoF) and 0.7–3.9° for knee (1 DoF) and ankle (2 DoF). RMSE for neck and trunk kinematics in [23]: 5.5–8.7°. MAE: ~20–30 mm (OpenPose) [14]. DeepLabCut ICC: 0.60–0.75+ [12]. Kinect: excellent for all joints in the anteroposterior (AP) direction (Pearson r ~ 0.98–0.99) and poor in the vertical (V) direction, especially for foot markers with Kinect v2 (r = 0.11–0.01) [13].	3D point cloud error: ~2 mm; The nominal accuracy (point-to-point difference) of 3D is around 0.2 mm [29]. Anthropometric AMAD: ~10 mm [36] Waist-to-hip ratio LoA: ±0.06 and Body-fat%: high agreement with BIA [42].
**Cost**	OMC: very high (specialized cameras and force plates). IMU: moderate.	Generally low (consumer-grade cameras).	Low (depth cameras, mobile phones).
**Portability**	OMC: Lab-bound. IMU: Portable.	Highly portable.	Moderate to high portability.
**Set-up Complexity**	Specialized laboratory setup.	Medium to high. Requires multi-camera calibration for 3D. Custom training may improve performance.	Medium. May require manual annotation, sufficient space for clear view volume, or assistance
**Validation Status**	OMC: Gold standard. IMU: Validated vs. OMC but inconsistencies exist.	Extensively validated vs. OMC. Promising agreement but needs further testing.	Validated vs. 3D scanners, BIA, DXA. Anthropometric validity still under refinement.
**Key Applications**	Clinical gait assessment, Rehabilitation, Obesity-related movement studies.	Movement analysis, Joint kinematics, Gait parameters, Home-based monitoring.	Anthropometric measurement, BF% estimation, Body shape modeling.

**Table 15:** SWOT Analysis of Optical and Markerless Motion Capture Technologies for Human Movement Assessment.

	Strengths	Weaknesses	Opportunities	Threats
**Optical Marker-Based Systems**	High accuracy and precision in both kinematic and kinetic analysis Gold standard benchmark for validation of other technologies	Expensive systems with high infrastructure requirements Time-consuming setup with detailed marker placement and calibration Marker influence on natural movement and soft tissue artifacts (STA), especially in obesity	Longitudinal studies in research labs where detailed ground-truth data are critical Development of improved STA mitigation techniques	Increasing accessibility and accuracy of markerless systems may reduce OMC adoption User burden and maintenance complexity limit scalability and portability
**Markerless Pose Estimation Systems**	Non-invasive, no physical contact needed More cost-effective and scalable than marker-based systems Can be used in uncontrolled, real-world environments Open-source and customizable (e.g., DeepLabCut, OpenPose)	Accuracy varies depending on pose complexity and occlusions Sensitive to camera placement and dataset generalizability Computationally intensive for large-scale or high-res video	Suitable for clinical screening in space-constrained clinics and homes Ideal for longitudinal ecological data collection and remote monitoring	Unreliable results in highly dynamic or occluded environments Rapid algorithmic changes may hinder long-term consistency in clinical pipelines
**3D Body Scanners (Camera-Based)**	Rapid and automated 3D body measurement High repeatability and convenience for users	Sensitive to acquisition noise and missing data Pose dependency: accuracy depends on maintaining a canonical pose	Monitoring obesity and body composition in clinical/home settings Studying longitudinal anthropometric trends in research labs	Measurement variations from small errors can reduce clinical trust Competing low-cost smart scales and wearables offer partial alternatives
**Hybrid Optical Approaches**	Improves robustness and accuracy by combining multiple camera views Reduces tracking errors and improves 3D reconstruction via fusion Enables detailed visual analysis under occlusions or diverse movements	Setup and data processing complexity increases Higher computational cost and manual intervention in complex worklows	Ideal for high-fidelity monitoring in complex or clinical populations Useful in visually challenging environments with occlusion risks	Limited accessibility due to multi-camera setup requirements Greater need for synchronization and calibration among optical components

**Table 16:** Summary of ethical dimensions in obesity-related gait and body modeling systems: technical approaches, privacy risks, and bias mitigation strategies.

Study/Source	Technical Approach	Privacy Impact	Bias Mitigation/Validation
Markerless System Development [19]	Multi-camera, open-source, DeepLabCut.	Not explicitly detailed as an impact but raises privacy questions, especially when considering wide deployment	Customizable models, open-source transparency.
Deep Learning and Thermal Imaging [31]	Near-Infrared (NIR) Spectroscopy and Infrared Thermal Imaging (IRT), deep learning (CNNs, transfer learning models like VGG-16/19).	Data not public Informed consent.	Stratified sample Automation for efficiency.
Depth Cameras for 3D Reconstruction [29].	Kinect/RealSense KinectFusion	Detailed body shape data	Controlled objects for validation, repeatability.
Body Shape Change Detection [46].	2D photos, image processing	Reluctance to upload images	Prototype models, call for more data.
Privacy-Preserving Gait Detection [9]	Encrypted optical system, ML	Focus on identity protection, encrypted skeletons	Privacy-preserving mechanisms.
Body Fat from 3D Kinect Scans [28].	Kinect v2, depth-maps, regression	Home use, self-scan; less manual processing	Acknowledges info loss, error awareness
Gait Assessment in Individuals with Obesity [10].	GAITRite, Matlab scripts	Not addressed	Confirms group differences, small sample size
Influence of BMI on Gait Metrics (Systematic Review) [5].	IMUs, AI algorithms (Random Forest, SVM)	Informed consent; Data contained within the article.	Small obese group, confounders, call for diversity
Reliability of OptoGait Photoelectric Cell System [8].	Photoelectric cell system	Informed consent; low privacy impact	Focus on reliability, power analysis
Depth Camera and IMU Integration [21].	Depth camera, IMUs, Vicon, BRANN	Not detailed	Alternative to Vicon, acknowledges IMU limits
Kinetic Program in Obese Children [11].	BTS G-WALK system (G-SENSOR inertial system, G-Studio software) for gait parameters and pelvic kinematics	High priority on safety and well-being of child participants (vulnerable population), confidentiality, parental consent	Notes single device limitations, reporting bias
Reliability and Validity of Gait Analysis using 3D Markerless Pose Estimation [34].	3D markerless pose estimation algorithms (OpenPose, 3DPoseNet); Single-camera video	Informed consent from participents; Raw data planned to be made available without undue reservation	Lower accuracy than marker-based Suggests training “networks that are specific to each population”
Multi-sensor Wearable System [56].	Multi-sensor wearable system (INDIP) with IMUs and force-resistive sensors; Stereophotogrammetric system as reference	Informed consent; public datasets	sensor redundancy limits wearability, validated across cohorts
Feature Hiding in 3D Scans [44].	3D scan data processing using Analogia Graph and feature shape templates; Surface rendering methods: blurring and transparency	Focus on the privacy of body parts Demonstrates how user preferences for privacy vary with security context	Tested on CAESAR, quantifies privacy preferences
DeepLabCut for Gait in Children [12].	DeepLabCut, 2D video	Informed consent, ethical compliance	Underexplored validity, arm swing occlusion
Detailed 3D Human Body Reconstruction from Multi-View Image [22].	Coarse-to-fine method combining 3D reconstruction from multi-view images (MF-PIFu) and Voxel Super Resolution (VSR) to infer detailed 3D human body models	Not directly detailed as a privacy concern but involves the creation of detailed 3D human body models which capture comprehensive body shape information.	Evaluates input views, compares to prior methods
Intel RealSense Camera for Measuring Health Outcomes [30].	Intel RealSense 3D depth sensing camera; Comparisons with Vicon 3D motion analysis system and GAITRite pressure pad system	As a review paper, it does not involve human participants. Notes that some traditional systems are expensive and confined to specialist centers, limiting widespread use	Supports the use of technology to develop robust, objective endpoints
Health&Gait Dataset [25].	Video sequences of participants walking; Optical flow computation; Machine learning for BMI, age, sex estimation	Informed consent for data sharing in an anonymized form that does not allow for identification	Stratified sampling by age, sex, and BMI
Kinect Cameras for Spatio-temporal Gait Parameters [13].	Kinect v2/Azure, Vicon as reference system	Not discussed	Validated vs. Vicon, algorithm error noted
Mobile Application (LeanScreen) for Body Composition [42].	2D digital photography (LS2D) and 3D photonic scanning (LS3D); Compared to conventional methods (Gulick tape, BIA, skinfolds, DXA)	No explicit concerns beyond image capture	Validity/comparison with conventional methods
Systematic Review of Technologies for Human Movement Analysis in Obese Subjects [20].	Marker-based optoelectronic stereophotogrammetric systems; Wearable MIMUs; Medical imaging for validation	Not discussed	Notes STA, gender/obesity stratification needed
Deep Learning-Based 3D Body Modeling from 2D Images [24]	3D generative model creating 3D body data from 2D images	No external data sharing; informed consent	Compared to 3D scanner, average error reported

**Table 17:** Overview of sample sizes, accuracy, and deployment contexts in obesity detection technologies.

Category	Sample Size	Accuracy Metrics	Deployment Context
**Single-Smartphone Video Pose Estimation/Inertial Sensors**	One study collected gait samples from 63 subjects using smartphone sensors [27]. Another study trained deep learning models on gait data from 138 participants (92 normal, 46 overweight/obese) and tested them on an additional 35 participants (23 normal, 12 overweight/obese) [6]. The Health&Gait dataset for video-based gait analysis includes 398 participants and 1564 videos [25]. For 2D image to 3D body data generation, training data from 400 subjects (200 male, 200 female) in their 20s and 30s were used, and validation was performed on 214 people (103 men, 111 women) in the same age group [24].	- BMI prediction: up to 96.54% accuracy with a Gaussian filter and KNN classifier [27]. - Age prediction: up to 96.19% accuracy using a square filter and SVM [27]. - Hybrid CNN-LSTM: 97% accuracy, Precision 0.99, Recall 0.9123, F1-score 0.9541 [6]. - Gait classification: 89.6% accuracy, 87.3% precision [5]. - 2D imaging: ±5.16% waistline, ±4.58% hip size [46]. - 2D→3D body data: 93% accuracy, 5.4–8.2% specific errors [24]. - Gait parameters: speed correlation 0.80; stride length correlation 0.11 [25]. - The mobile application LeanScreen (LS2D): bias 0.5%, LoA −6.8% to 7.8% [42].	- Easy-to-use, accessible BMI and age prediction. - Instant gait pattern recording. - Self-photography for anthropometric measures. - Non-contact sensor system, continuous body monitoring.
Compact RGB-D Scanners	- 38 participants (body fat, Kinect v2) - 19 volunteers (waistline measurement) - Mannequins/cylinders (3D accuracy test) - 5 subjects (treadmill gait) - 240 adults (LeanScreen LS3D)	- Body fat from Kinect v2: RMSE 8.02% (adj-R2 = 0.72) - Kinect V2 vs. RealSense: Kinect 4.60 ± 1.25 mm accuracy; RealSense 7.59 ± 1.66 mm; 2.0 mm difference = 2.0 cm girth variation - Azure Kinect: step length RMSE 0.02; Kinect v2: 0.03 - LeanScreen LS3D: BF% overestimation bias 3.4–4.8%, WHR high agreement, LoA −0.06 to 0.06	- Low-cost 3D body scanning - Single depth map and skeletal tracking for home use - Portable platforms for in-clinic/field assessment - Telemedicine, telerehabilitation - OR-compatible alternatives - Convenient, non-invasive home body tracking systems

**Table 18:** Real-world deployment status of reviewed optical sensor-based methods.

Method/Technology	Deployment Status	Key Notes
Marker-based motion capture	Clinical/research standard	High accuracy, costly, lab-bound
Markerless video-based (OpenPose, MediaPipe)	Pilots, research studies	Accessible, ongoing validation, limited robustness
RGB-D cameras (Kinect, RealSense)	Clinics, research, some home use	Portable, moderate cost, expanding clinical adoption
Smartphone-based gait analysis	Pilot studies, emerging commercial apps	High accessibility, validation ongoing
AI/deep learning models	Research, early clinical pilots	Rapid evolution, needs explainability and clinical validation
